# AUTS2 expression within mammalian lineage: A predictor of neural networks involved in autism spectrum disorders

**DOI:** 10.1016/j.gendis.2024.101440

**Published:** 2024-10-19

**Authors:** Aude-Marie Lepagnol-Bestel, Yann Loe-Mie, Mounia Bensaid, Michel Simonneau

**Affiliations:** a INSERM U894, Centre de Psychiatrie et Neuroscience, Université Paris Descartes, Paris 75014, France; b Université de Reims Champagne-Ardenne, URCATech, 2 Av. Robert Schuman, Reims 51100, France; c Ecole Royale de Service de Santé Militaire. Hôpital Militaire d'Instruction Mohammed V, Rabat 10100, Morocco; d Université Paris-Saclay, École Normale Supérieure Paris-Saclay, Centre National de la Recherche Scientifique, CentraleSupélec, LuMIn, Gif-sur-Yvette 91190, France; e Department of Biology, École Normale Supérieure Paris-Saclay Université Paris-Saclay, Gif-sur-Yvette 91190, France

The autism susceptibility candidate 2 (AUTS2) gene^1^^,^^2^ at 7q11.2 was first identified and found disrupted because of a balanced translocation in a pair of monozygotic twins with autism spectrum disorder (ASD). Analysis of 60 novel cases suggests that clinical phenotypes are more closely associated with intellectual disability rather than directly linked to ASD features. Human *AUTS2* is a highly conserved gene that spans 1.2 Mb. Human AUTS2 protein has two major isoforms, full-length (1259 aa) and C-terminal (711 aa). Phenotypic analysis of patients indicated that they had borderline to severe intellectual disability/developmental delay, and 83%–100% had microcephaly. Mild dysmorphology was present. Specific traits of autism (like obsessive behavior) were seen frequently (83%). *AUTS2* is also associated with alcohol consumption, heroin dependence, schizophrenia, and dyslexia, as analyzed using GWAS studies.

Our working hypothesis is that the analysis of the distribution of *AUTS2* during mammalian evolution can predict behavioral phenotypes in the different animal models. Here, we analyzed the distribution of AUTS2 transcripts in mice, marmosets, and humans, during brain development and the evolution of *AUTS2* locus by comparing sequences from Neanderthal, Denisovan, and modern humans.

To understand what phenotypes in animal models could be related to human AUTS2 syndrome, we took advantage of recent mouse public databases of *in situ* hybridization (ISH): GenePaint (https://gp3.mpg.de/) for embryonic and adult brain (Allen Brain Atlas at www.mouse.brain-map.org). The open Marmoset Gene Atlas (https://gene-atlas.brainminds.jp/) established a genome-wide atlas of the gene expression of *Callithrix jacchus* brain. We also generated quantitative radioactive ISH data from human embryos. *AUTS2* was shown to be implicated in human evolution, with several regions where its human sequence significantly changed when compared with Neanderthals and non-human primates.^3^ We used sequences from Neanderthal, Denisovan, and modern humans to analyze the evolution of transcription factor binding sites in these regions. Altogether, we cover the different branches, including mouse, marmoset, Neanderthal, Denisovan, and modern humans, that appeared from their ∼90 MY common ancestor (Fig. 1A; Fig. S1).

**Figure 1:**
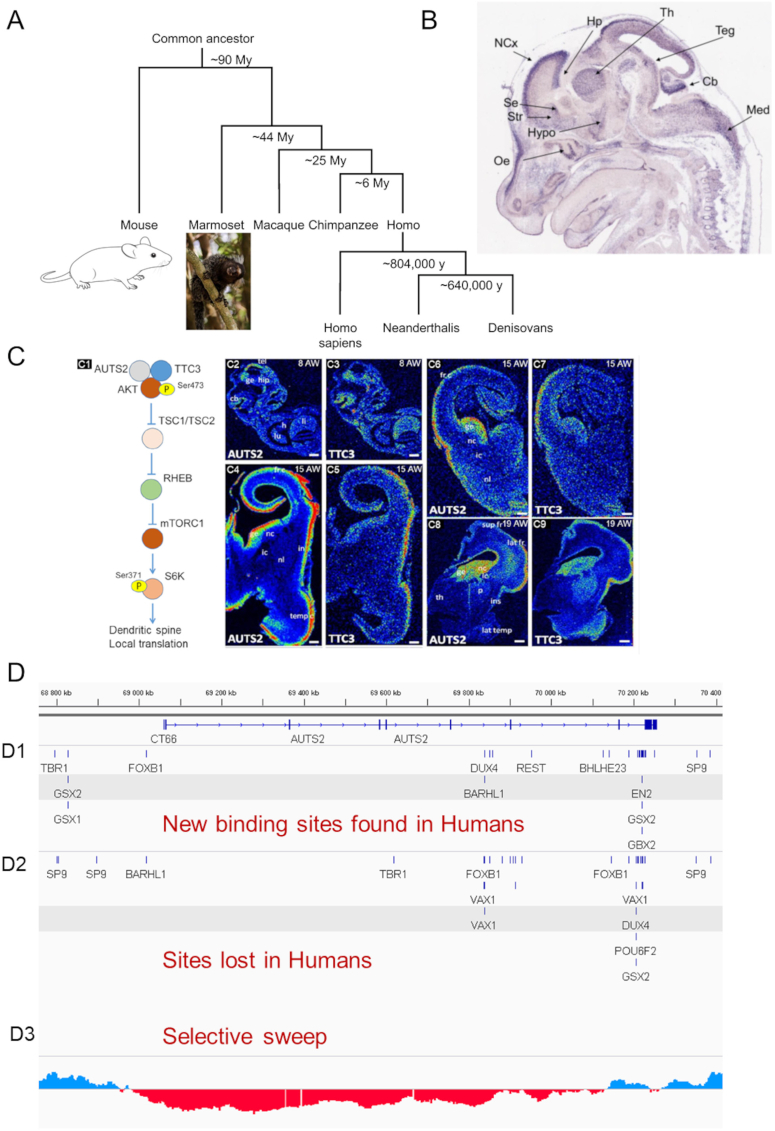
Expression of *AUTS2* gene in different mammals predicts phenotypes found in patients. **(A)** DNA sequences of the Neanderthals and the Denisova hominins were estimated to have diverged on average 640,000 years ago, and from present-day Africans around 804,000 years ago (Reich et al, 2010). Specimens are not drawn to scale. **(B)** By *in situ* hybridization, *Auts2* expression is observed in multiple areas of the mouse brain on embryonic day 14.5 (E14.5), including the neocortex (Ncx), hippocampus (Hp), dorsal thalamus (Th), septum (Se), striatum (St), olfactory epithelium (Oe), hypothalamus (Hypo), tegmentum (Teg), cerebellum (Cb), and medulla (Med). Sagittal section from Genepaint (https://gp3.mpg.de/). **(C)***AUTS2* and *TTC3* mRNA levels in the human central nervous system during early development and at mid-gestation. (C1) mTORC1 pathway that involves AUTS2-TTC3-AKT complex (modified from Lepagnol-Bestel et al, 2022). (C2, C3) Sagittal sections of 8-week-old human embryos hybridized with *AUTS2* (C2) and *TTC3* (C3) antisense radioactive riboprobes. The two transcripts were detected in the telencephalon (tel), ganglionic eminence (ge), hippocampus anlagen (hipp), cerebellum anlagen (Cb), and liver (li) but not in the heart (h) and lung (lu). (C4–C7) Coronal sections of 15-week-old human brains hybridized with *AUTS2* (C4–C6) and *TTC3* (C5–C7) antisense radioactive riboprobes. The two transcripts were detected in the frontal cortex (frc), temporal cortex (tempc), insular cortex (in), and germinal zone (ge) but not in the nucleus caudate (nc), nucleus lenticular (nl), and internal capsule (ic). (C8, C9) Coronal sections of 19-week-old human brains hybridized with *AUTS2* (C8) and *TTC3* (C9) antisense radioactive riboprobes. The two transcripts were detected in the frontal superior cortex (supfr), lateral frontal cortex (latfr), insular cortex (ins), ganglionic eminence (ge), internal capsule (ic), and nucleus caudate (nc) but not in the thalamus (th), putamen (p), and lateral temporal cortex (lattemp). Scale bars = 1 mm. **(D)** Human *AUTS2* gene displays changes in brain-specific transcription factor binding sites in modern humans as compared with Neanderthal and Denisova hominins. Genomes (hg19) from extinct hominins and ancient humans were obtained from the Max Planck Institute for Evolutionary Anthropology, Leipzig, Germany. Genomic data of the chromosome 7 locus including the *AUTS2* gene (1,790,971 bp; chr7:68,765,409–70,556,380) are visualized using the UCSC Genome browser. 33 new binding sites found in modern humans (D1) and 46 binding sites lost in modern humans (D2) were respectively identified. The last track (D3) shows the Neandertal selective sweep Z-score (negative red values indicate a selective sweep in ancient humans).

In mice, *Auts2* expression is observed in multiple areas of the mouse brain on embryonic day 14.5, including the neocortex, hippocampus, dorsal thalamus, septum, striatum, olfactory epithelium, hypothalamus, tegmentum (in particular in substantia nigra and ventral tegmental area), cerebellum, and medulla oblongata (Fig. 1B; Fig. S2). In the adult mice, higher expression was found in dentate gyrus, cornu ammonis 3, subiculum, lateral entorhinal cortex, temporal association areas, and cerebellum (Fig. S3). The septum, hypothalamus, and cerebellum are known to be involved in social communication. The prefrontal cortex and striatum are implicated in stereotypies and perseverative behaviors. The prefrontal cortex, dentate gyrus, cornu ammonis 3, subiculum, and lateral entorhinal cortex participate in memory and cognition. The prefrontal cortex, striatum, substantia nigra, and ventral tegmental area are part of the reward circuit linked to alcohol consumption. The dentate gyrus, cornu ammonis 3, subiculum, and lateral entorhinal cortex are involved in recognition memory linked to dyslexia.

Recently, the open Marmoset Gene Atlas has published AUTS2 expression results in the brain. In the neonatal brain, *AUTS2* expression can be visualized in the prefrontal cortex, particularly in Brodmann area 24 (A24; anterior cingulate cortex), A6, and A8, and in movement-control related areas (Caudate, Putamen, and Thalamus) (Fig. S4). The prefrontal cortex, basal ganglia, and thalamus are structures linked to cognition and movement control, respectively, known as impaired in ASD.

We next analyzed the expression of *AUTS2* at three stages of human brain development: 8, 15, 18, and 22 weeks (Fig. 1C; Fig. S5, 6). We used radioactive antisense riboprobes. *AUTS2* expression was quantified by optical imaging of the spatial distribution of beta-particles emerging from brain sections. We have recently demonstrated that AUTS2 directly interacts with tetratricopeptide repeat domain 3 (TTC3), an E3 ligase of AKT that regulates dendritic spine function via mTORC1 (mechanistic target of rapamycin complex 1)-dependent local translation. In 8-week-old human embryos hybridized with AUTS2 and TTC3 riboprobes, we found the two transcripts in the telencephalon, ganglionic eminence, hippocampus anlagen, and cerebellum anlagen. In 15-week-old brains, AUTS2 and TT3 were co-expressed in the frontal cortex, temporal cortex, insular cortex, and germinal zone (Fig. 1C). In 19-week-old human brains, AUTS2 and TT3 were co-expressed in the frontal superior cortex, lateral frontal cortex, insular cortex, GE, internal capsule, and nucleus caudate (Fig. 1C). In 15-week-old human embryos, *AUTS2* was expressed in the frontal cortex, hippocampus, temporal cortex, insula and ganglionic eminence anatomically subdivided into the medial, lateral, and caudal. All medial, lateral, and caudal ganglionic eminence expressed *AUTS2*. These transient structures generate distinct populations of interneurons. From these patterns of co-expression, one expects to have phenotype changes in cognition (frontal cortex and cortical interneurons from medial ganglionic eminence), in learning & memory (hippocampus anlagen and hippocampal interneurons from ganglionic eminence), in the social brain (frontal cortex, insula, and cerebellum) and stereotypies and perseverative behaviors (caudate, striatum interneurons from ganglionic eminence). Expression of *AUTS2* in interneurons is important as interneurons display innovation in human lineage.^4^

We next examined binding sites for brain-specific transcription factors in the AUTS2 locus to test if they varied between extinct hominins and modern humans. We obtained genomic data from the Max Planck Institute for Evolutionary Anthropology, Leipzig, Germany, and visualized using the UCSC Genome Browser (Fig. 1D). One high-coverage (∼30-fold coverage) Denisovan genome, three Neanderthal genomes (2 high coverage >20x and a low coverage), and three ancient human genome sequences were used. We selected sites if the 3 ancient humans were similar to the Human hg19 references, the Denisovan genome was mutated, and at least 2 Neanderthals out of three were mutated for the base. From this criterion, we selected 171 sites from which we extracted short sequences of 31 bp (15 bp before the mutated site, 15 bp after) one for humans (hg19) and one for ancient hominins. Our analysis was focused on brain-specific transcription factors. Altogether, we identified 33 new binding sites found in modern humans and 46 binding sites lost in modern humans (Fig. 1D and Table S1–S3). Furthermore, we found that *AUTS2* enhancers were modified from hominins to modern humans (Fig. S7–S10). We found 3 enhancers classified as “distal enhancer-like signature” overlapping with novel FOXB1 (forkhead box B1), FERD3L (Fer3 like BHLH transcription factor), and REST (RE1 silencing transcription factor) binding sites present in modern humans and not in hominids. We evidenced an increase in gained sites for *TBR1* (T-box brain transcription factor 1) and *EN2* (engrailed homeobox 2). *TBR1* is characterized by its expression that defines molecularly distinct domains within the cerebral cortex.^5^ Mouse *En2* mutants impact cerebellum development and mesencephalon dopaminergic neurons, defects relevant to human neurodevelopmental disorders in particular ASD. We identified subtypes of neurons and neuronal networks where AUTS2 levels correlate with novel transcription factors found in modern humans as compared to archaic hominins (Fig. S11–17). In particular, we identified a a ∼20 kb AUTS2 sequence able to reveal functional impacts of AUTS2 transcriptional changes in modern humans as compared to archaic hominins (Fig. S16). Finally, we studied the correlation of AUTS2 levels with transcription factors in marmoset and mouse brains (Fig. S18–20).

Structures linked to social communication, stereotypies, perseverative behaviors and memory & cognition can be identified in mouse. Importance of interneurons and temporo-spatial changes in expression pattern of AUTS2 between ancient hominins and *Homo sapiens* point to subtle modifications of these behaviors.

Altogether, these results suggest that ISH distribution along mammals is a novel phenotype-related biomarker useful for translational research.

## Author contributions

**Aude-Marie Lepagnol-Bestel:** Conceptualization, Investigation. **Yann Loe-Mie:** Data curation, Formal analysis. **Mounia Bensaid:** Investigation. **Michel Simonneau:** Conceptualization, Funding acquisition, Writing – review & editing.

## Ethics declaration

For human embryonic and fetal brain tissues used in this manuscript, research was approved by the French Ministry for Research (IE-2014-760; validated on September 5, 2014).

## Funding

This work was founded by SFR Cap Santé, Université de Reims Champagne-Ardenne, Reims, France to Aude-Marie Lepagnol-Bestel, a European Grant ERA-Net Cofund Action on Nanomedicine under Horizon 2020 Euronanomed 3 (project MoDiaNo), and CNES (Centre National d'Etudes Spatiales) grant (MemoBion) to Michel Simonneau.

## Conflict of interests

The authors declare that they have no competing interests.
