# Supplementary material for: AUTS2 expression within mammalian lineage: A predictor of neural networks involved in autism spectrum disorders
Source: Genes Dis. 2024 Oct 19;12(3):101440. doi: 10.1016/j.gendis.2024.101440 (PMC11786819; doi:10.1016/j.gendis.2024.101440)
Supplement: Multimedia component 1 [file mmc1.docx]

***Rapid communication supplemental data***

Title: Auts2 expression within mammalian lineage: a predictor of neural networks involved in autism spectrum disorders

## Phylogenetic data from mouse to modern humans

The autism susceptibility candidate 2 (AUTS2) gene on chromosome 7q11.22 at 7q11.2 was first identified and found disrupted as a result of a balanced translocation event (t7;20) ^1^ in a pair of monozygotic twins with autism spectrum disorder (ASD). Identification of more than 60 novel cases suggest that clinical phenotypes of AUTS2 related patients are more closely associated with intellectual disability (ID) rather than directly linked to classical features associated with ASD. Human *AUTS2* is a highly conserved gene that spans 1.2Mb. Human AUTS2 protein has two major isoforms, full-length (1259 aa) and C-terminal (711 aa). This short C-terminal protein is produced from an alternative transcription start site in exon 9. An AUTS2-Polycomb complex was shown to activate gene expression in the CNS ^2^.

Human *AUTS2* display nucleotide variants that define the human-Neanderthal sweep ^3^ and three human accelerated regions (HARs) ^4,5^.

Phenotypic analysis of AUTS2 syndrome patients was recently performed ^6^. All patients have borderline to severe ID/developmental delay, 83–100% have microcephaly. Mild dysmorphology are present. Behaviour is marked by a friendly outgoing social interaction. Specific traits of autism (like obsessive behaviour) are seen frequently (83%), but classical autism was not diagnosed in any.

In most of the cases, one can find small in-frame deletions which are often inherited and give a mild clinical phenotype associated with. Deletions and other mutations causing haploinsufficiency of the full length *AUTS2* transcript, that occur *de novo*, give a more severe phenotype. Previous studies have shown deletions within the C-terminus isoform spanning exons 9-19 are associated with a severe neurocognitive phenotype ^7^. AHDH was found in a patient was a partial duplication of 3’ part of *AUTS2* locus ^8^. ASDs have been described for full deletions and duplications of the *AUTS2* locus ^9^. Furthermore, diverse GWAS studies found that *AUTS2* locus is associated to a variety of neurological conditions such as addiction disorders ^10,11^, epilepsy ^12^, schizophrenia ^13,14^ and dyslexia ^15,16^.

To understand what phenotypes in animal models can be related to human AUTS2 syndrome ^6,7,17^, we took advantages of recent public databases of large-scale *In Situ* Hybridation (ISH) performed in two mammalian neurobiology models, mouse and marmoset. GenePaint is a digital atlas of gene expression patterns in various tissues and species with strong focus on mouse embryos ([https://gp3.mpg.de/).](https://gp3.mpg.de/)) Expression patterns are determined by non-radioactive ISH on serial tissue sections. Allen Brain Atlas is an anatomically comprehensive digital atlas containing the expression patterns of
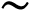
20,000 genes in the adult mouse brain [(www.mouse.brain-map.org)](http://www.mouse.brain-map.org/). Recently, the open Marmoset Gene Atlas [(https://gene-atlas.brainminds.jp/)](https://gene-atlas.brainminds.jp/) established a genome-wide, high-resolution atlas of the gene expression throughout the common marmoset (Callithrix jacchus) brain. It uses ISH analysis to systematically analyze changes in gene expression over the course of postnatal brain development to adult stage ^18,19^.

We also generated quantitative radioactive ISH data from human embryos as in ^20^. *AUTS2* was also shown to be implicated in human evolution, having several regions where its human sequence significantly changed when compared to Neanderthals and non-human primates. We used sequences from Neanderthal, Denisovan and modern human to analyze evolution of transcription factor binding sites in these regions.

Altogether, we cover the different branches, including mouse, marmoset, Neanderthal, Denisovan and modern human, that appeared from their ~ 90 MY common ancestor (**Sup. Fig. 1**).

***Supplemental figure 1. Phylogenetic tree relating the mouse, Marmoset, Denisova hominins, Neanderthal and Homo sapiens.****DNA sequences of the Neanderthals and the Denisova hominins were estimated to have diverged on average 640,000 years ago, and from present-day Africans around 804,000 years ago. Specimens are not drawn to scale.*


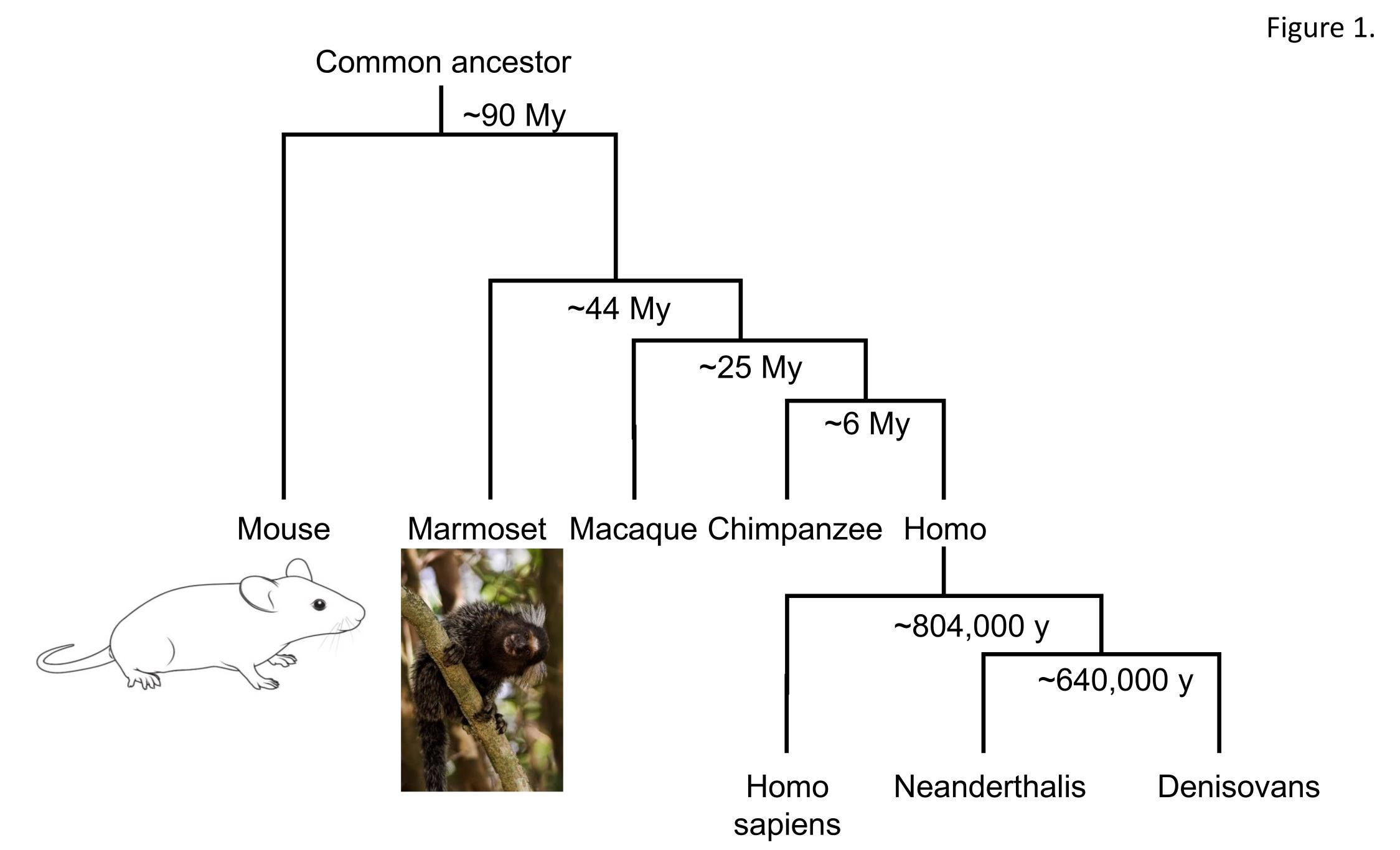


Neanderthals

## Auts2 expression in developing and adult mouse brain

We took advantage of public databases to analyze developing mouse brain (Genepaint [(https://gp3.mpg.de/))](https://gp3.mpg.de/)) and adult mouse brain (Allen Brain Atlas at [www.mouse.brain-map.org)](http://www.mouse.brain-map.org/).). *Auts2* expression is observed in multiple areas of E14.5 mouse brain, including Neocortex, Hippocampus, Dorsal thalamus, Septum, Striatum, Olfactory epithelium, Hypothalamus, Tegmentum, Cerebellum and Medulla oblongata (**Sup. Fig. 2**).


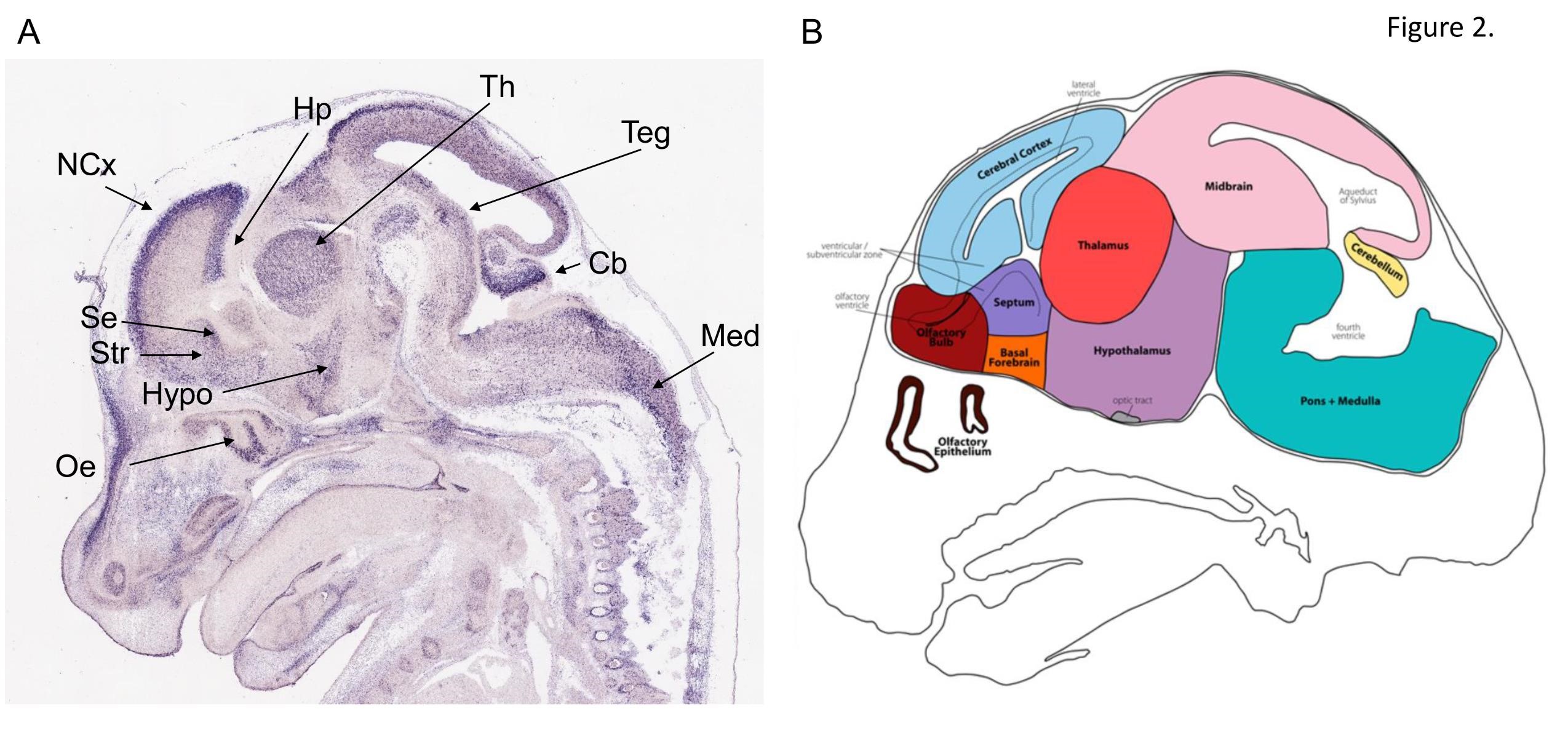


***Supplemental figure 2.*** ***AUTS2 expression in developing mouse brain.***

*A. By in situ hybridization, Auts2 expression is observed in multiple areas of E14.5 mouse brain, including Neocortex (Ncx), hippocampus (Hp), dorsal thalamus (Th), Septum (Se), Striatum (St), Olfactory epithelium (Oe), Hypothalamus (Hypo), Tegmentum (Teg), cerebellum (Cb), and medulla (Med). Sagittal section from Genepaint (https://gp3.mpg.de/).*

*B. Schematic of main brain regions for E14.5 mouse (from GeneSat) (http://www.gensat.org).*

Interestingly, concerning the expression of *Auts2* in the tegmentum, it was demonstrated that virtually all of the dopaminergic Tyrosine Hydroxylase positive neurons, that are located in tegmentum, in particular in Subtantia Nigra (SN) and Ventral Tegmental area (VTA) express *Auts2* ^21^. Interestingly, SN and VTA are located at the posterior region of the Tegmentum, at the junction between Midbrain and Pons (**Sup. Fig. 2B**).

By ISH, *Auts2* expression is observed in multiple areas of the adult brain (C57BL/6J strain; 56 days of age; male) on a coronal section (**Sup. Fig.3A**). Higher expression is found in Dentate Gyrus (DG), Cornu Ammonis 3 (CA3), Subiculum, Lateral Entorhinal Cortex and temporal association areas. Visual area, Auditory Cortex and Cortical amygdalar area, posterior part medial zone express *Auts2* (**Sup. Fig. 3A**). Furthermore, in adult mouse Cerebellum (sagittal section), *Auts2* transcripts localized in the layer of Purkinje cells (**Sup. Fig. 3B**). In the E14.5 mouse brain, *Auts2* expression was homogeneous along the rostrocaudal axis. However, by E16, the Auts2 expression pattern was reported to change with an expression limited to superficial layers ^21^.


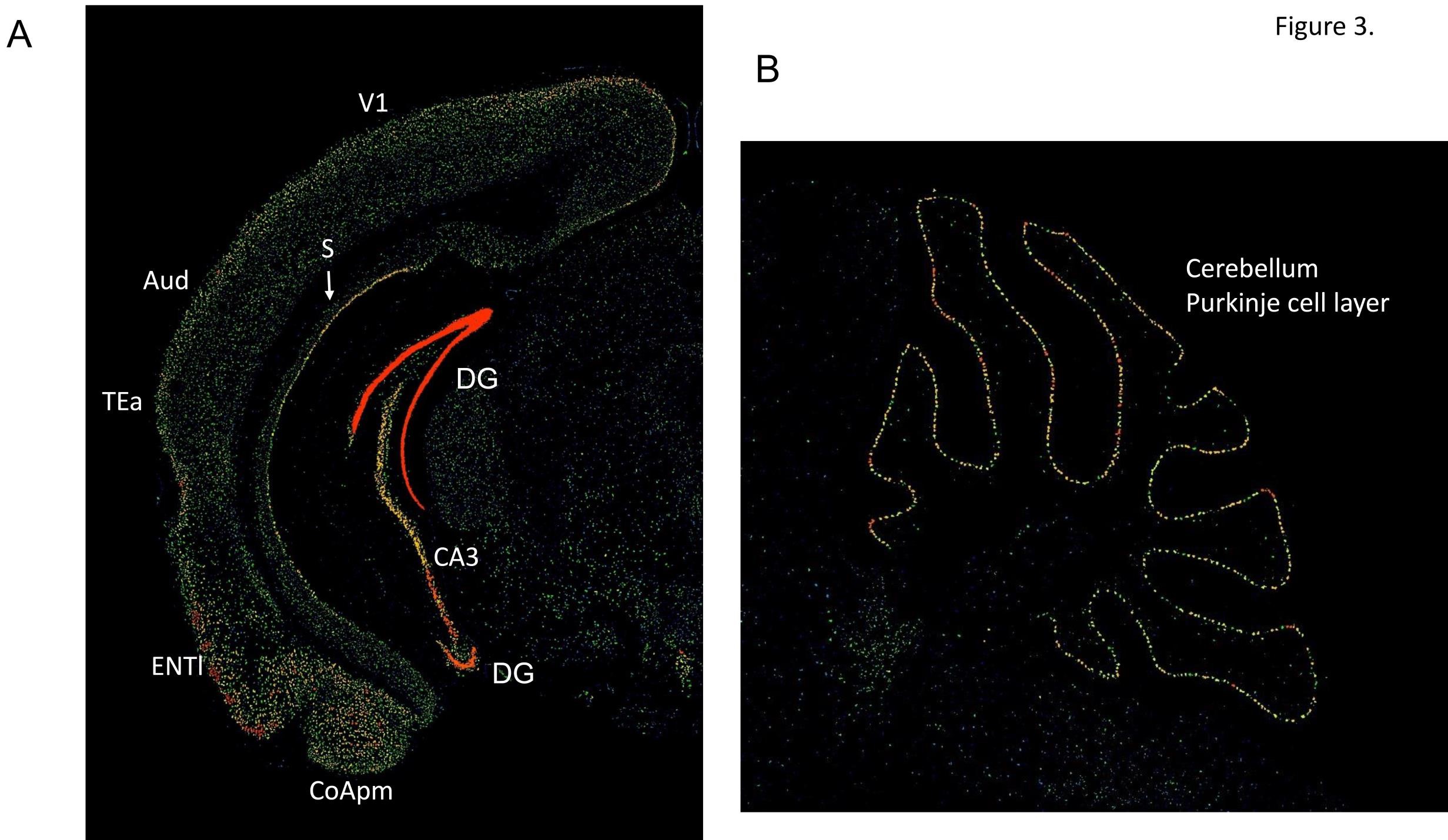


***Supplemental figure 3. AUTS2 expression in adult mouse brain***

*A. By in situ hybridization, Auts2 expression is observed in multiple areas of the adult brain; on a coronal section. Higher expression is found in Dentate Gyrus (DG), Cornu Ammonis 3 (CA3), Subiculum (Sub), Visual area (VI), Auditory Cortex (Aud), Temporal association areas (TEa), Entorhinal area, lateral part (ENTl) and Cortical amygdalar area, posterior part medial zone (COApm).*

*B. In adult mouse cerebellum (sagittal section), Auts2 transcripts localized in the layer of Purkinje cells.*

*Data from Allen Brain Atlas.*

These patterns of expression are in full agreement on previous report of *Auts2* expression ^21,22^.

From these patterns of expression, it is possible to correlate Auts2-expressing neuronal networks with phenotypes related to ASDs, IDs, alcohol consumption or dyslexia. Hippocampus, Septum, Hypothalamus and Cerebellum are known to be involved on social communication ^23^. Prefrontal Cortex and Striatum are implicated in stereotypies and perseverative behaviors ^24^. Prefrontal Cortex, DG, CA3, Subiculum and Lateral Entorhinal Cortex participate to memory and cognition ^25^. Prefrontal Cortex, Striatum, SN and VTA are part of the reward circuit linked to alcohol consumption ^26^. DG, CA3, Subiculum and Lateral Entorhinal Cortex are involved in recognition memory that is linked to dyslexia ^27,28^.

# *AUTS2 expression results in the developing and adult non-human primate brain: the marmoset*

Recently, the open Marmoset Gene Atlas ([https://gene-atlas.brainminds.jp/)](https://gene-atlas.brainminds.jp/) has published AUTS2 expression results in the developing and adult non-human primate brain. In the marmoset neonatal brain, *AUTS2* expression can be visualized in the Prefrontal Cortex, in particular in Brodmann area 24

(A24; Anterior Cingulate Cortex), A6 and A8 (**Sup. Fig. 4A-B**) and in movement-control related areas (Caudate, Putamen, Thalamus) (**Sup. Fig. 4A-B; Sup. Fig. 4C**). Other regions with high *AUTS2* expression are cortical regions receiving sensory afferences: Piriform Cortex, Orbital Proisocortex (Opro) and Gustatory Cortex (**Sup. Fig. 4A**). Interestingly, *AUTS2* is also express in the Claustrum (**Sup. Fig. 4A**).

Hippocampus and related regions including Lateral Entorhinal Cortex and Temporal Cortex express *AUTS2* (**Sup. Fig. 4C; Sup. Fig 4E**). High levels of AUTS2 are also found in Amygdala (**Sup. Fig. 4C-E**) and in all layers of Lateral Geniculate Nucleus (LGN) (**Sup. Fig. 4D; Sup. Fig. 4F**).

Hippocampus and related regions can be involved in memory defects. Interestingly, CA2 that is known as a critical hub of sociocognitive memory processing ^29,30^, highly expressed *AUTS2*. Fronto-striatal pathways are involved in stereotypies and perseverative behaviors ^24^. Expression in Amygdala suggests possible implication in anxiety and fear associative memory ^26,31^. The claustrum is a brain region that has been investigated for over 200 years but its precise function remains unknown ^32^. Sir Francis Crick with Christof Koch suggested that the claustrum can be critically linked to consciousness ^33^. Widespread extensive connectivity of single claustrum neurons with the entire cerebral cortex suggests a prominent role in higher order processes ^34^.

Expression in LGN can be related to the involvement of the visual magnocellular pathway in ASDs ^35,36^.

In adult marmosets, *AUTS2* mRNA levels remain high only in the Amygdala, and in Hippocampal DG.


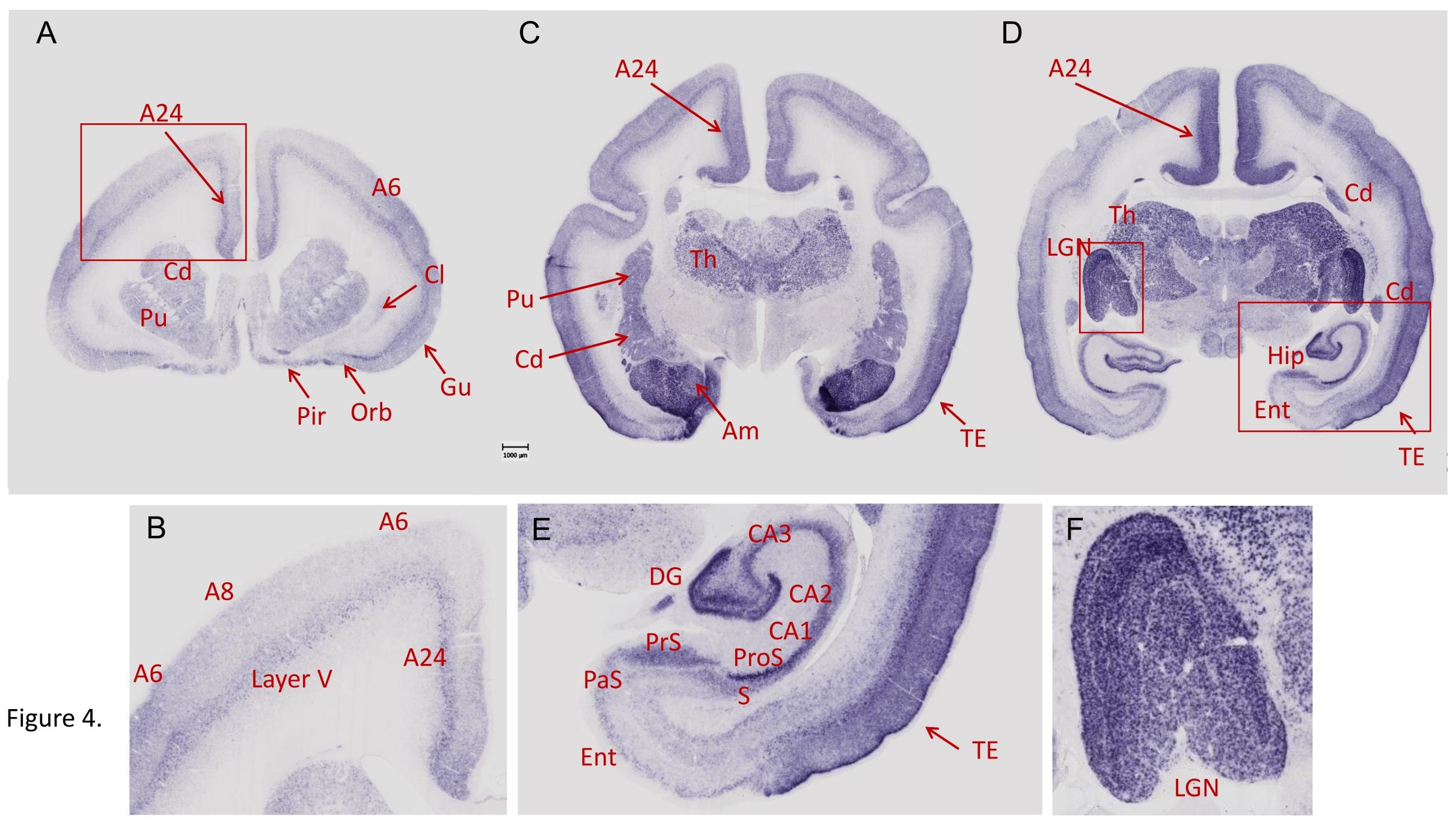


***Supplemental figure 4. AUTS2 expression in neonate marmoset brain.***

*A, C and D sections are coronal sections from anterior to posterior axis.*

*A. The highest levels of AUST2 are detected in Brodmann A24 (anterior cingulate cortex), caudate (Cd), putamen (Pu), claustrum (Cl), Brodmann A24 (anterior cingulate cortex), A6 and A8 that are all part of the frontal cortex, Piriform cortex (Pir), Orbital Proisocortex (Opro) and Gustatory cortex (Gu).*

*B. Enlargement of the Brodmann A24 (anterior cingulate cortex) (red frame in A). Expression of AUTS2 is found in Brodmann A24 (anterior cingulate cortex), Brodmann A6 and A8, that all are part of the frontal cortex. Note that expression is higher in layers V for the Brodmann A24 (anterior cingulate cortex) and Brodmann A6, A8 areas.*

*C. The highest levels of AUST2 are detected in Brodmann A24 (anterior cingulate cortex), Thalamus (Th), caudate (Cd), putamen (Pu) , Amygdala (Am) and Temporal cortex area (TE).*

*D. The highest levels of AUST2 are detected in Brodmann A24 (anterior cingulate cortex), Temporal cortex (TE), caudate (Ca), Thalamus, Hippocampus and related structures and Lateral Geniculate Nucleus (LGN).*

*E. Enlargement of the Hippocampus and related structures (red frame in D). Hippocampus: Dentate Gyrus (DG), Cornu Ammonis 1 (CA1), Cornu Ammonis 2 (CA2), Cornu Ammonis 3 (CA3), Subiculum (S) Presubiculum (PrS), Prosubiculum (ProS) and Parasubiculum (PaS). Entorhinal cortex (Ent) and Temporal cortex area (TE). Note that expression is higher in layer V of the TE.*

*F. Enlargement of the LGN region (red frame in D). Note that expression of AUTS2 is found in all layers of LGN (magnocellular, parvocellular and koniocellular layers).*

*Data from the Marmoset Gene Atlas (*[*https://gene-atlas.brainminds.riken.jp/*](https://gene-atlas.brainminds.riken.jp/)*).*

## AUTS2 expression analyzed in the developing human brain

We analyzed expression of *AUTS2* at three stages of human brain development: 8, 15, 18 and 22 weeks of amenorrhea respectively (**Sup. Fig. 5; Sup. Fig.6**). We used radioactive antisense riboprobes. *AUTS2* expression was quantified by optical imaging of the spatial distribution of beta-particles emerging from brain sections ^20,37^.


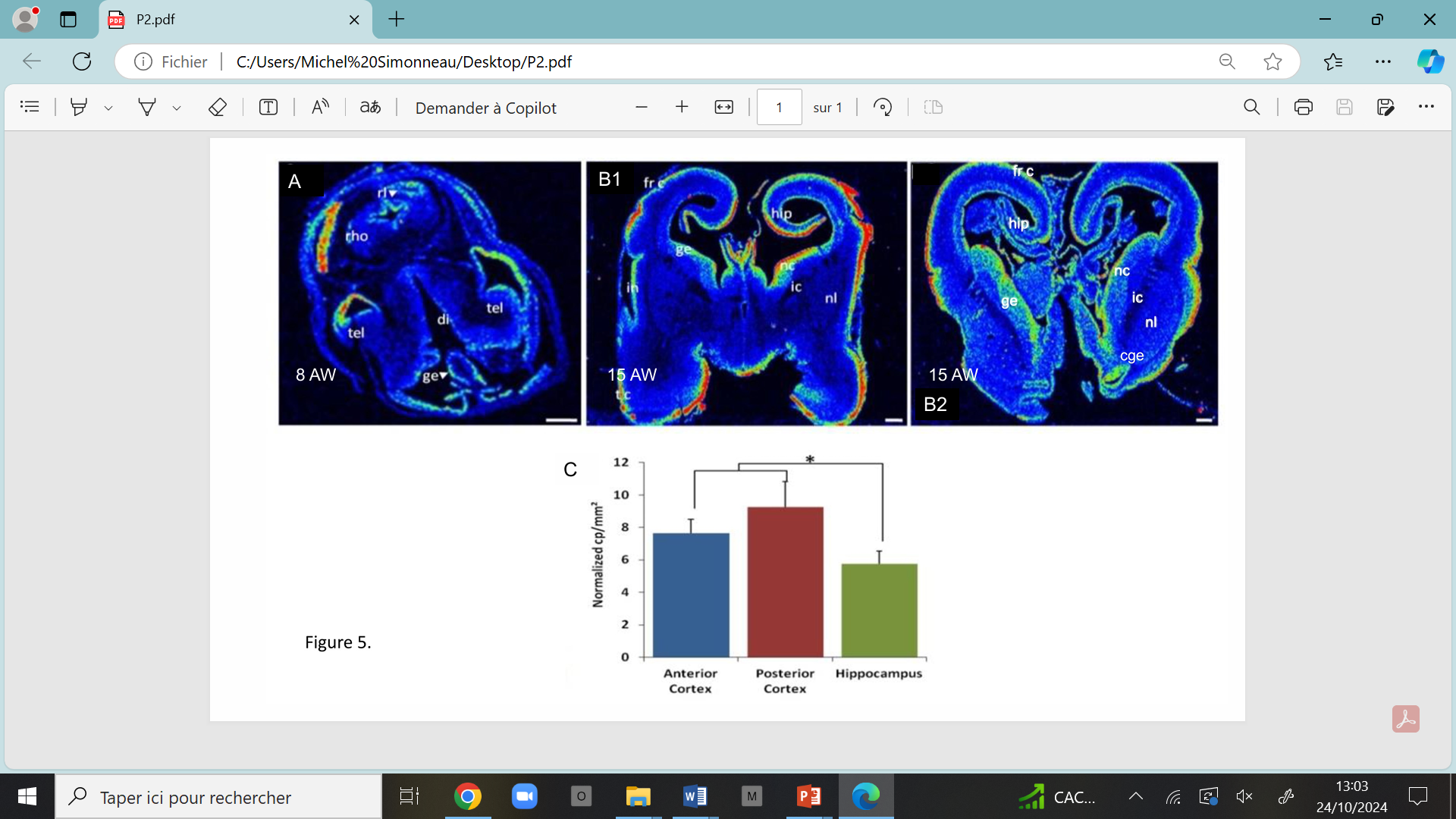


***Supplemental figure 5.*** ***AUTS2 expression during human brain development***

*A-B. Coronal sections of 8-week (A) and 15-week-old (B) human embryos (B1 anterior section and B2 posterior section) hybridized with AUTS2 antisense radioactive riboprobe.*

*di: diencephalon; ic: internal capsule; fr c: frontal cortex; ge: germinal zone; hip: hippocampus; in: insular cortex; mes: mesencephalon; nc: nucleus caudate; nl: nucleus lenticular; rho: rhombencephalon; rl: rhombic lip; tc: temporal cortex; tel: telencephalon.*

*Scale bar=1mm.*

*C. quantification indicating expression of AUTS2 both in cortex and hippocampus at 15-weekold human embryonic development with a significantly higher expression level in cortical regions than in hippocampus. * = p<0.05*

In 8-week human embryo (**Sup. Fig 5A**), *AUTS2* is expressed in Rhombencephalon; rhombic lip and germinal eminence. Ganglionic eminences (GE) are subcortical structures of gray matter which appear during the 5^th^ week post-fertilization on the floor of telencephalic vesicles ^38^.

In 15-week-old human embryos (**Sup. Fig 5B**; B1 anterior section and B2 posterior section), *AUTS2* is expressed in Frontal Cortex, Hippocampus, Temporal Cortex, Insula and GE. The GE is anatomically subdivided into the medial (MGE), lateral (LGE) and caudal (CGE). At this stage, both MGE and LGE ganglionic eminence express *AUTS2* as visualized on an anterior coronal (frontal) section in **Sup. Fig.** **5B1**. On a posterior coronal section (**Sup. Fig. 5B2**), AUST2 expression can be visualized in MGE, LGE and CGE. These transient structures generate main neuronal networks. The MGE and LGE develop into the basal ganglia, striatum and pallidum respectively ^39,40^. The LGE generates projection neurons to the striatum, the medium spiny neurons that form 90% of the neuronal striatum population. The CGE gives rise to Amygdala ^39,40^. GE also generate a variety of interneurons. From the LGE, interneurons migrate to the Olfactory Bulb. The CGE produces interneurons migrating to the Cerebral Cortex ^41,42^. The MGE is a main source of interneurons throughout the Cortex, Hippocampus and Striatum after tangential migration ^43,44^.

Quantification of *AUTS2* expression both in Cortex and Hippocampus at 22-week-old human embryonic development with a significantly higher expression level in cortical regions than in Hippocampus (p<0.05) (**Sup. Fig. 5C**).

We recently demonstrated that AUTS2 directly interacts with TTC3 a E3 ligase of AKT that regulates dendritic spine function via mTORC1-dependent local translation ^45^ (**Sup. Fig. 6A**). In 8-week-old human embryos hybridized with AUTS2 and TTC3 antisense radioactive riboprobes, we found the two transcripts in Telencephalon, GE, Hippocampus anlagen and Cerebellum anlagen (**Sup. Fig. 6B1-B2**). In 15week-old human brains, AUTS2 and TT3 are co-expressed in Frontal Cortex, Temporal Cortex, Iinsular Cortex and germinal zone but nor in nucleus caudate (nc), nucleus lenticular (nl) and internal capsule (ic) (**Sup. Fig. 6C1-C2, Sup. Fig. 6D1-D2**). In 19-week-old human brains AUTS2 and TT3 are co-expressed in Frontal Superior Cortex, Lateral Frontal Cortex, Insular Cortex, GE, Internal Capsule and Nucleus Caudate (**Sup. Fig. 6E1-E2**).

From these patterns of co-expression, one expects to have phenotype changes in cognition (Frontal Cortex and cortical interneurons from MGE), in learning & memory (Hippocampus anlagen and hippocampal interneurons from GE), in social brain (Frontal Cortex, Insula and Cerebellum) and stereotypies and perseverative behaviors (caudate, striatum interneurons from GE).


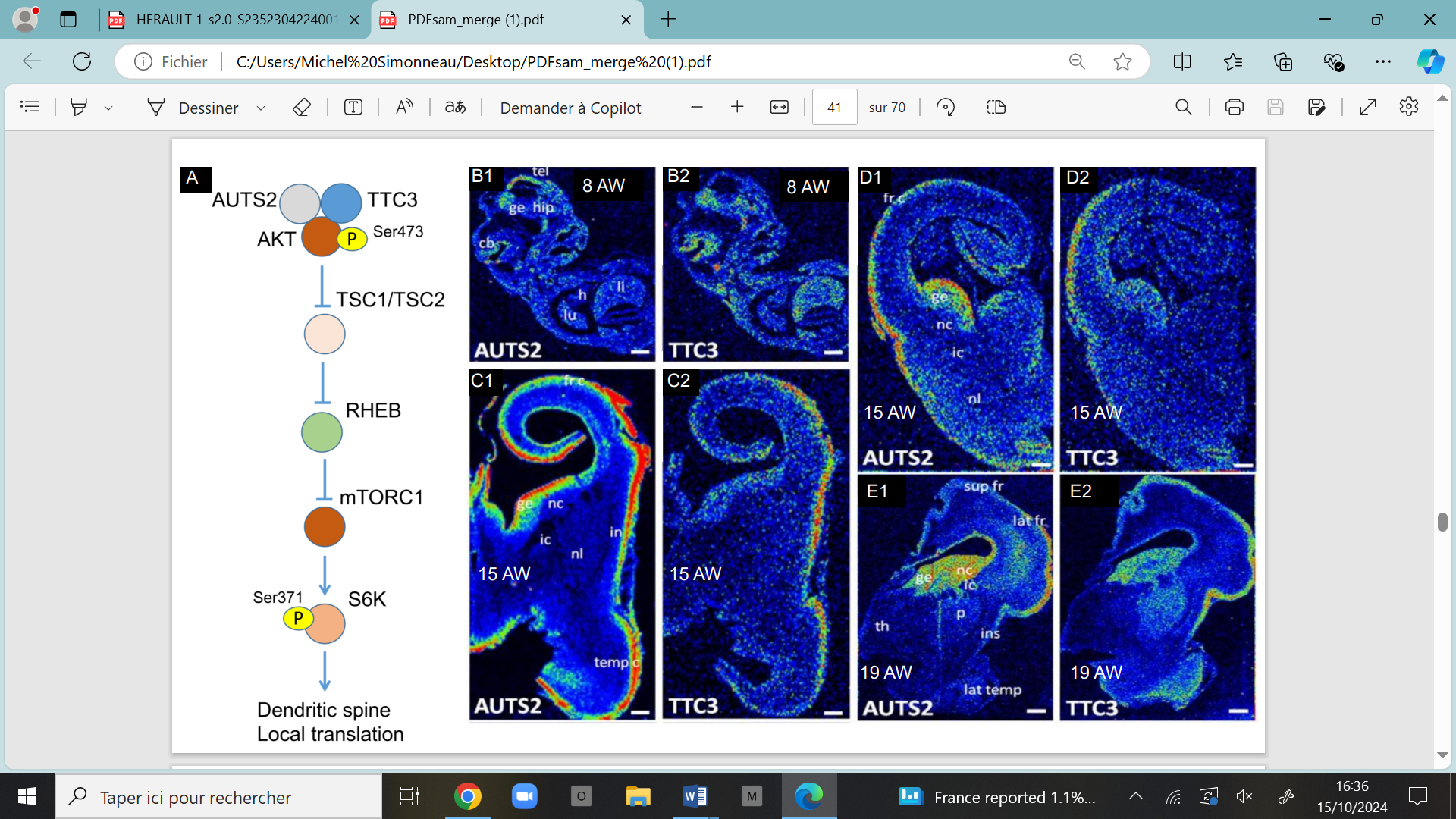


***Supplemental figure 6.*** ***AUTS2 and TTC3 mRNA levels in the human central nervous system during early development and at mid-gestation***

*A. mTORC1 pathway that involves AUTS2-TTC3-AKT complex (modified from Lepagnol-Bestel et al., 2022).*

*B. Sagittal sections of 8-week-old human embryos hybridized with AUTS2 (B1) and TTC3 (B2) antisense radioactive riboprobes. The two transcripts were detected in telencephalon (tel), ganglionic eminence (ge), hippocampus anlagen (hipp), cerebellum anlagen (Cb) and liver (li) but not in heart (h) and lung (lu).*

*C-D. Coronal sections of 15-week-old human brains hybridized with AUTS2 (C1-D1) and TTC3 (C2-D2) antisense radioactive riboprobes. The two transcripts were detected in frontal cortex (fr c), temporal cortex (temp c), insular cortex (in) and germinal zone (ge) but nor in nucleus caudate (nc), nucleus lenticular (nl) and internal capsule (ic).*

*E. Coronal sections of 19-week-old human brains hybridized with AUTS2 (E1) and TTC3 (E2) antisense radioactive riboprobes. The two transcripts were detected in frontal superior cortex (sup fr), lateral frontal cortex (lat fr), insular cortex (ins), ganglionic eminence (ge), internal capsule (ic) and nucleus caudate (nc) but not in thalamus (th), putamen (p) and lateral temporal cortex (lat temp).*

*Scale bars=1mm.*

## Binding sites for brain-specific transcription factors in the AUTS2 locus varied between extinct hominins and modern humans

Our working hypothesis was that binding sites for brain-specific transcription factors in the *AUTS2* locus varied between extinct hominins and modern humans.

Mutations in coding sequences between hominins and modern humans induce changes in neurogenesis ^46,47^. Similar changes are expected for mutations in binding sites for brain-specific transcription factors in the *AUTS2* locus. We used genomes (hg19) from extinct hominins and ancient humans obtained from Max Planck Institute for Evolutionary Anthropology, Leipzig, Germany.

<http://cdna.eva.mpg.de/neandertal/Vindija/VCF/>

Genomic data of the chromosome 7 locus including the *AUTS2* gene (Human hg19: 1,790,971 bp; chr7: 68,765,409-70,556,380) were visualized using the UCSC Genome browser (**Sup. Fig. 7**). We used genotypes for the Vindija, Altai and Denisova genomes generated using snpAD, from an ancient DNA damage-aware genotyper developed in Leipzig for the analysis of the Vindija genome. It uses an empirical error profile that captures the position-dependent probabilities of base exchanges due to error and deamination to estimate the proportion of all possible genotypes by maximum likelihood.

One high-coverage (~30-fold coverage) Denisovan genome was sequenced from bones recovered in Denisova Cave in the Altai Mountains in southern Siberia and were dated to 72,000 years ^48^.

Three Neanderthal genomes were used. The first one is a high-coverage (> 20x coverage) Neanderthal genome also sequenced from bones recovered in Denisova Cave in the Altai Mountains in southern Siberia and dated to 122,000 years ^49^.The second one is the genome of an infant (*Mezmaiskaya 1*) indirectly dated to around 70–60,000 years old, recovered in Mezmaiskaya Cave of the [North Caucasus](https://en.wikipedia.org/wiki/North_Caucasus) sequenced to a low genomic coverage ^49^. The third sample is the genome of a female Neandertal from ~50,000 years ago from Vindija Cave, Croatia, sequenced to ~30-fold genomic coverage ^50^.

We also used three ancient human genome sequences. The first one is the Linearbandkeramik (LBK) sample, also referenced as ‘Stuttgart’ (19-fold coverage), from a
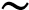
7,000-year-old skeleton found in Germany ^51^. The second one is the ‘Loschbour’ sample (22-fold), an
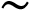
8,000-year-old skeleton from the Loschbour rock shelter in Luxembourg, discovered in the context of hunter-gatherer artefacts ^51^. The third ancient human genome sequence is a high-quality (42-fold sequence coverage) sequence of a ~45,000-year-old modern human male from Siberia (Ust_Ishim) ^52^.


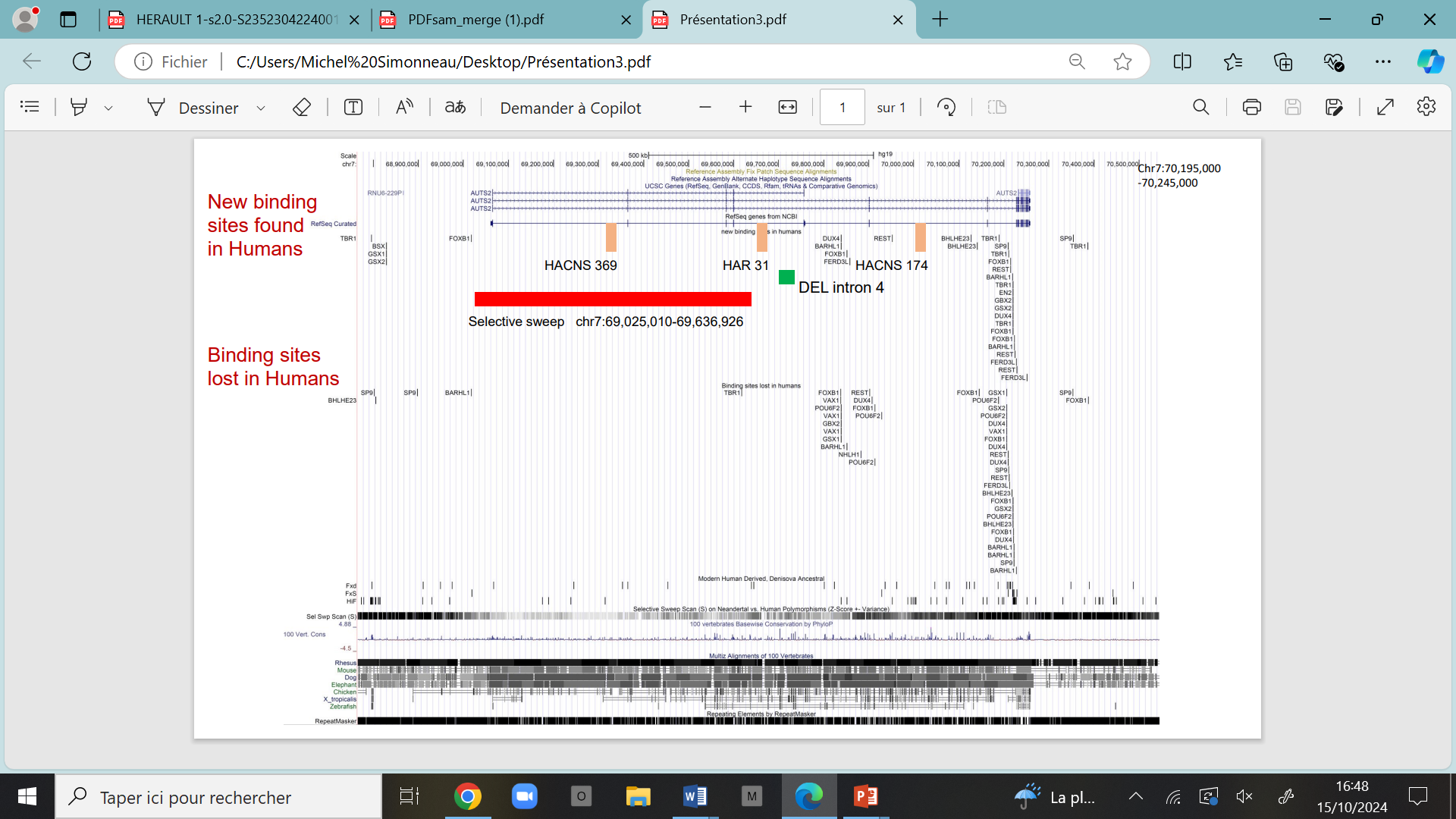


***Supplemental figure 7.*** ***Human AUTS2 gene displays changes in brain-specific transcription factors binding sites in modern humans as compared to Neanderthal and Denisova hominins***

*Genomes (hg19) from extinct hominins and ancient humans were obtained from Max Planck Institute for Evolutionary Anthropology, Leipzig, Germany. Genomic data of the chromosome 7 locus including the AUTS2 gene (1,790,971 bp; chr7: 68,765,409-70,556,380) are visualized using the UCSC Genome browser. In orange (not to scale) human accelerated sequences are indicated (HACNS 369, HAR 31 and HACNS 174). Note that these elements do no express new transcription factor binding sites. In red, the human Neanderthal selective sweep is indicated from 69,025,010 to 69,636,926.*

*In the AUTS2 locus, we identified 33 new sites in modern humans and not present in archaic hominins and 46 sites lost in modern humans and present in archaic hominins.*

*Fxd = fixed in modern human (ancestral Denisovan) (>99%)*

*FxS = fixed but in dbSNP (>99%)*

*HiF = High Frequency (> 90%)*

We selected sites if the 3 ancient humans were similar to the Human hg19 references, the Denisovan genome was mutated and if at least 2 Neanderthals out of three were mutated for the base. From this criterion, we selected 171 sites. From these sites, we extracted short sequences of 31bp (15bp before the mutated site, 15bp after) one for humans (hg19) and one for ancient hominins. We performed Position Weight Matrix search scans with MOODS-DNA ^53^ and the human matrixes from the JASPAR database ^54^. We focused our analysis on sites of transcription factors that are specific for brain ^55^. Altogether, we identified 33 new binding sites found in modern humans and 46 binding sites lost in modern humans (**Sup.Table 1**) (**Sup. Fig. 7**).

Sites lost in modern humans

|  | start | end Site |
| --- | --- | --- |
| chr7 | 68802161 | 68802191 SP9 |
| chr7 | 68805206 | 68805236 BHLHE23 |
| chr7 | 68897125 | 68897155 SP9 |
| chr7 | 69017263 | 69017293 BARHL1 |
| chr7 | 69617531 | 69617561 TBR1 |
| chr7 | 69837645 | 69837675 FOXB1 |
| chr7 | 69837645 | 69837675 VAX1 |
| chr7 | 69838308 | 69838338 POU6F2 |
| chr7 | 69838308 | 69838338 VAX1 |
| chr7 | 69838308 | 69838338 GBX2 |
| chr7 | 69838439 | 69838469 VAX1 |
| chr7 | 69838439 | 69838469 GSX1 |
| chr7 | 69851321 | 69851351 BARHL1 |
| chr7 | 69881797 | 69881827 NHLH1 |
| chr7 | 69901615 | 69901645 REST |
| chr7 | 69907011 | 69907041 DUX4 |
| chr7 | 69913573 | 69913603 POU6F2 |
| chr7 | 69913573 | 69913603 FOXB1 |
| chr7 | 69928556 | 69928586 POU6F2 |
| chr7 | 70145059 | 70145089 FOXB1 |
| chr7 | 70187813 | 70187843 POU6F2 |
| chr7 | 70205488 | 70205518 GSX1 |
| chr7 | 70205488 | 70205518 GSX2 |
| chr7 | 70205488 | 70205518 POU6F2 |
| chr7 | 70205488 | 70205518 DUX4 |
| chr7 | 70205488 | 70205518 VAX1 |
| chr7 | 70205692 | 70205722 FOXB1 |
| chr7 | 70205692 | 70205722 DUX4 |
| chr7 | 70209471 | 70209501 REST |
| chr7 | 70209507 | 70209537 DUX4 |
| chr7 | 70210613 | 70210643 SP9 |
| chr7 | 70211663 | 70211693 REST |
| chr7 | 70211849 | 70211879 FERD3L |
| chr7 | 70217204 | 70217234 BHLHE23 |
| chr7 | 70219588 | 70219618 FOXB1 |
| chr7 | 70219698 | 70219728 GSX2 |
| chr7 | 70219698 | 70219728 POU6F2 |
| chr7 | 70219767 | 70219797 BHLHE23 |
| chr7 | 70220883 | 70220913 FOXB1 |
| chr7 | 70220883 | 70220913 DUX4 |
| chr7 | 70221566 | 70221596 BARHL1 |
| chr7 | 70221580 | 70221610 BARHL1 |
| chr7 | 70222039 | 70222069 SP9 |
| chr7 | 70226874 | 70226904 BARHL1 |
| chr7 | 70352252 | 70352282 SP9 |
| chr7 | 70387180 | 70387210 FOXB1 |

Novel sites lost in modern humans

|  | Start | End | SITE |
| --- | --- | --- | --- |
| chr7 | 68795226 | 68795256 | TBR1 |
| chr7 | 68828576 | 68828606 | BSX |
| chr7 | 68828576 | 68828606 | GSX1 |
| chr7 | 68828576 | 68828606 | GSX2 |
| chr7 | 69017263 | 69017293 | FOXB1 |
| chr7 | 69838308 | 69838338 | DUX4 |
| chr7 | 69838308 | 69838338 | BARHL1 |
| chr7 | 69851321 | 69851351 | FOXB1 |
| chr7 | 69857973 | 69858003 | FERD3L |
| chr7 | 69952246 | 69952276 | REST |
| chr7 | 70126179 | 70126209 | BHLHE23 |
| chr7 | 70140489 | 70140519 | BHLHE23 |
| chr7 | 70188361 | 70188391 | TBR1 |
| chr7 | 70208919 | 70208949 | SP9 |
| chr7 | 70210613 | 70210643 | TBR1 |
| chr7 | 70214041 | 70214071 | FOXB1 |
| chr7 | 70214600 | 70214630 | REST |
| chr7 | 70219256 | 70219286 | BARHL1 |
| chr7 | 70219588 | 70219618 | TBR1 |
| chr7 | 70219609 | 70219639 | EN2 |
| chr7 | 70219609 | 70219639 | GBX2 |
| chr7 | 70219609 | 70219639 | GSX2 |
| chr7 | 70219767 | 70219797 | DUX4 |
| chr7 | 70220026 | 70220056 | TBR1 |
| chr7 | 70220026 | 70220056 | FOXB1 |
| chr7 | 70222612 | 70222642 | FOXB1 |
| chr7 | 70222727 | 70222757 | BARHL1 |
| chr7 | 70224022 | 70224052 | REST |
| chr7 | 70226874 | 70226904 | FERD3L |
| chr7 | 70228909 | 70228939 | REST |
| chr7 | 70251351 | 70251381 | FERD3L |
| chr7 | 70353421 | 70353451 | SP9 |
| chr7 | 70386005 | 70386035 | TBR1 |

***Sup. Table 1. TF sites lost (n=46) and gained (n=33) in modern humans as compared to archaic hominins***

Furthermore, the number of gained/lost brain-specific transcription factors binding sites in humans as compared to extinct hominins varied (**Sup. Fig. 8**). We evidenced an increase in gained sites for *TBR1* and *EN2*. *TBR1* is characterized by its expression that defines molecularly distinct domains within the cerebral cortex ^56^. Tbr1 transcription factor directly binds *Auts2* promoter and activates *Auts2* gene ^57^. Furthermore, neonatal Tbr1 dosage controls cortical layer 6 connectivity ^58^. Conditional *Tbr1* deletion during late mouse gestation in cortical layer 6 neurons (*Tbr1*^layer6^ mutants) gave novel insights into its function, including dendritic patterning, synaptogenesis, and cell-intrinsic physiology. These phenotypes occur in heterozygotes and may underlie ASD pathophysiology ^58^. Mouse *En2* mutants impact cerebellum development ^59^ and mesencephalon dopaminergic neurons ^60^, defects relevant to human neurodevelopmental disorders in particular ASDs.

Supplemental figure 8. Human AUTS2 gene displays changes in brain-specific transcription factors binding sites in humans as compared to Neanderthal and Denisova hominins

A. Histogram of gained/lost brain-specific transcription factors binding sites in humans as compared to Neanderthal and Denisova hominins. New sites identified in humans are indicated in blue; lost sites identified in humans are indicated in yellow.

B.C. Expression pattern of Tbr1 and En2 that display novel sites in in humans as compared to Neanderthal and Denisova hominins. Note that Tbr1 is expressed in neocortex, olfactory bulb and in ventral thalamus in E14 mouse brain. En2 is expressed in mesencephalon and cerebellum anlagen in E14 mouse brain. Sagittal sections from Genepaint (https://gp3.mpg.de/).
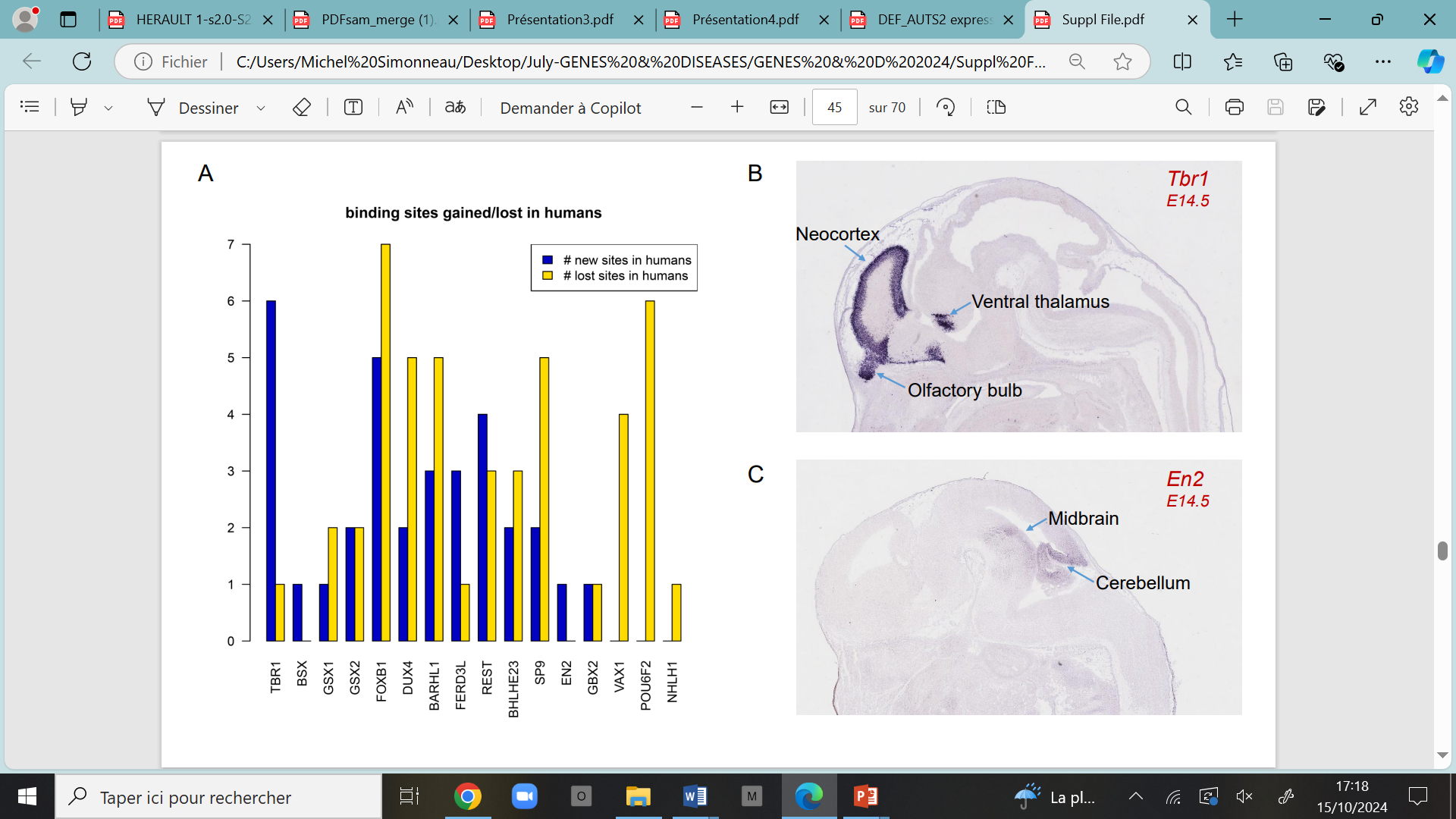


***Supplemental figure 8. Human AUTS2 gene displays changes in brain-specific transcription factors binding sites in humans as compared to Neanderthal and Denisova hominins***

*A. Histogram of gained/lost brain-specific transcription factors binding sites in humans as compared to Neanderthal and Denisova hominins. New sites identified in humans are indicated in blue; lost sites identified in humans are indicated in yellow.*

*B.C. Expression pattern of Tbr1 and En2 that display novel sites in in humans as compared to Neanderthal and Denisova hominins. Note that Tbr1 is expressed in neocortex, olfactory bulb and in ventral thalamus in E14 mouse brain. En2 is expressed in mesencephalon and cerebellum anlagen in E14 mouse brain. Sagittal sections from Genepaint (*[*https://gp3.mpg.de/*](https://gp3.mpg.de/)*).*

In contrast, *Vax1*, *Pou6f2* and *Nhlh1* brain-specific transcription binding sites are lost between extinct hominins and humans (**Sup. Fig. 8**). Using DropViz suite ^61^, we determined brain-cell clusters with highest expression for *Pou6f2* and *Nhlh1* adult mouse brain. The highest expression of *Pou6f2* is located in neurons of entopedoncular nucleus and subthalamic nucleus, gabaergic neurons of Substantia Nigra (SN). The highest expression of *Nhlh1* is located in Cajal-Retzius neurons of Hippocampus and in a neuronal cluster linked to neurogenesis in Hippocampus (**Sup. Fig. 9**).


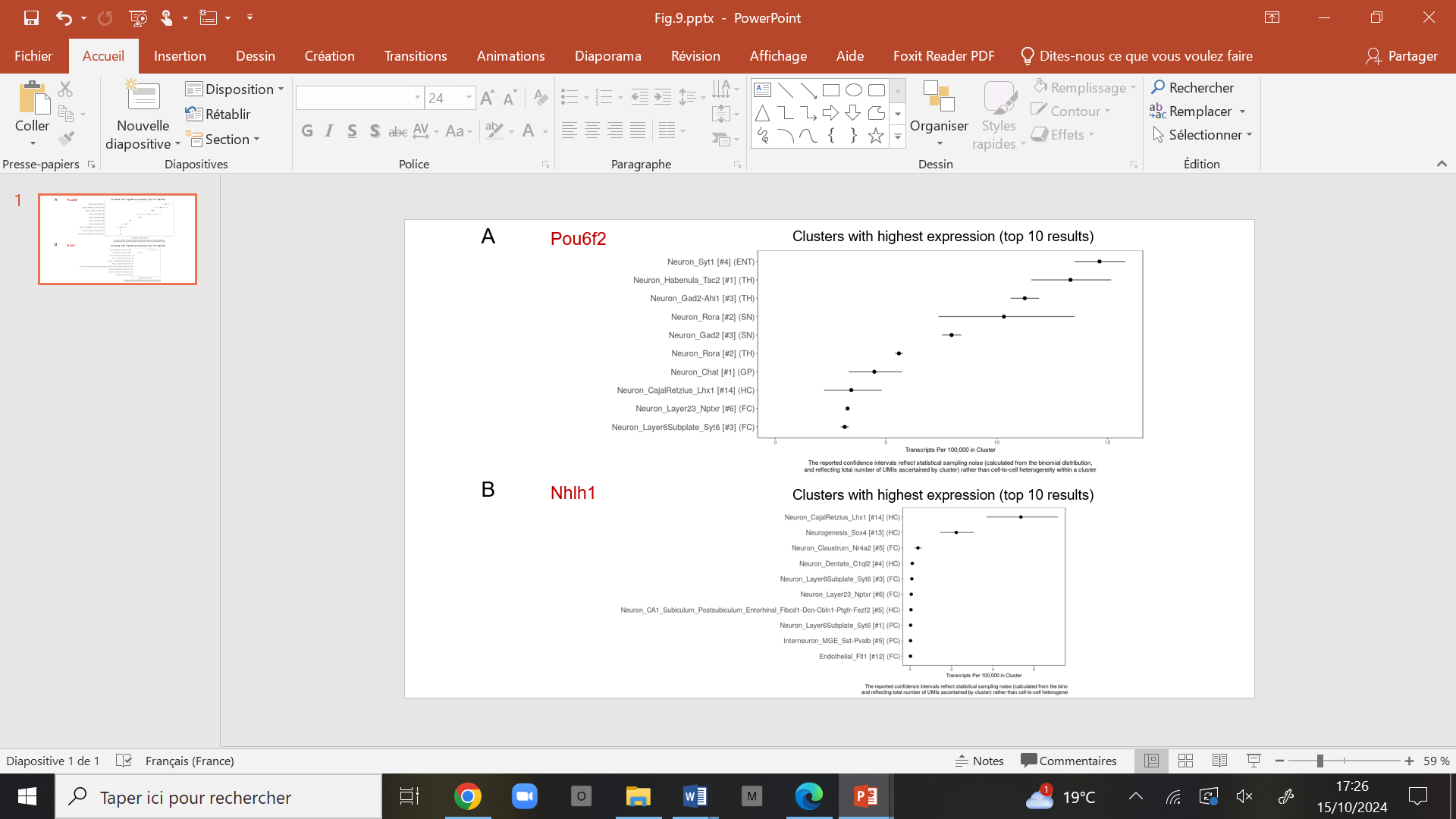


***Supplemental figure 9. Brain cell clusters with highest expression for genes encoding brain-specific transcription factors lost between extinct hominins and humans.***

*Vax1, Pou6f2 and Nhlh1 brain-specific transcription binding sites are lost between extinct hominins and humans. Using DropViz suite (Saunders et al., 2018), we determined brain-cell clusters with highest expression for Pou6f2 and Nhlh1 adult mouse brain.*

*The highest expression of Pou6f2 is located in neurons of entopedoncular nucleus and subthalamic nucleus (ENT), Thalamus (TH), gabaergic neurons of Substantia Nigra (SN). The highest expression of Nhlh1 is located in Cajal-Retzius neurons of hippocampus (HP) and in a neuronal cluster linked to neurogenesis in hippocampus (HP). Globus pallidus externus & nucleus basilis (GP), Frontal cortex (FC), Posterior cortex (PC).*

Altogether, these results suggest that changes in the *AUTS2* locus induced modifications of temporo-spatial regulation of AUTS2 expression between ancient hominids and modern humans, in particular in regions linked to brain diseases.

We previously found that REST (RE1 Silencing Transcription Factor) ^62^ regulates SMARCA2 and other genome-wide supported schizophrenia-associated genes ^63^. We evidenced a reorganization of REST sites for the *AUTS2* locus with 4 new REST-binding sites found in humans 3 lost REST-binding sites lost in humans (**Sup. Fig. 7**).

***Human AUTS2 locus: Identification of 33 novel sites found in modern humans and not in archaic hominins***


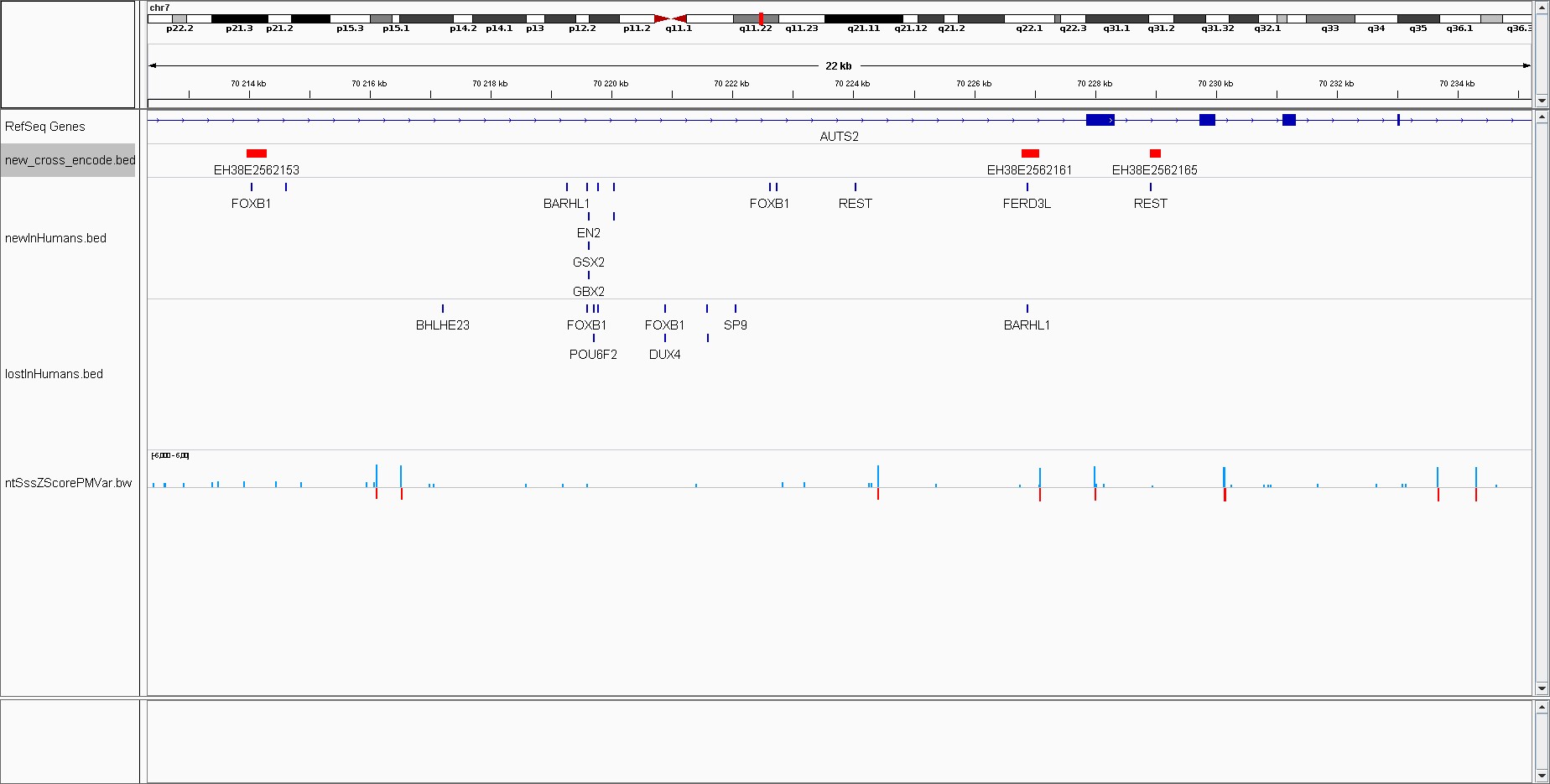
We identified 33 novel sites found in modern humans and not in archaic hominins (**Sup. Table 1 and Sup. Fig. 7**). With histone marks-based ENCODE database that is used in UCSC genome browser, we identified 3 enhancers classified as ENCODE “distal enhancer-like signature”: EH38E2562153, EH38E2562161, EH38E2562165 overlapping with FOXB1, FERD3L and REST binding sites, respectively that are part of the 33 sites that we identified as novel sites found in modern humans and not in archaic hominins (**Sup. Fig. 10**). They are qualified of distal enhancer-like defined by a signature of high DNase and H3K27ac signal. They are denoted distal due to their presence outside 2 kb of an annotated GENCODE transcription start site.

***Supplemental figure 10. Human AUTS2 gene displays changes in sequences of enhancers inducing changes in brain specific transcription factors binding sites in modern humans as compared to Neanderthal and Denisova hominins.***

*We took the ENCODE Candidate Cis-Regulatory Elements (cCREs) from the UCSC genome browser website (at UCSC Genome Browser Home) and intersected with our “new in humans” transcription factors binding sites.*

*We found 3 enhancers classified as “distal enhancer-like signature”: EH38E2562153, EH38E2562161, EH38E2562165 overlapping with FOXB1, FERD3L and REST binding sites, respectively.*

We next asked if these 33 sites can be validated by two different methods whose results are available at <https://chip-atlas.org/>^64^, the first based on protein-DNA interaction (TF) and the second, on the detection of accessible chromatin regions based on their increased accessibility to Tn5 transposase integration (ATACseq) ^65^. Interestingly, 6 sites (2*TBR1*, 1*BARHL1*, 1*FOXB1*and 2*SP9*) out of the 33 identified in our study are identified by the three techniques (Sup. Table 2).

| **hg19** |  | **Start** | | **End Novel site TF_overlap ATAC_overlap** | | | |
| --- | --- | --- | --- | --- | --- | --- | --- |
| chr7 |  | 68795226 | | 68795256 TBR1 | |  | TBR1 |
| chr7 |  | 68828576 | | 68828606 BSX | | BSX |  |
| chr7 |  | 68828576 | | 68828606 GSX1 | | GSX1 |  |
| chr7 |  | 68828576 | | 68828606 GSX2 | | GSX2 |  |
| chr7 |  | 69017263 | | 69017293 FOXB1 | |  | FOXB1 |
| chr7 |  | 69838308 | | 69838338 DUX4 | |  |  |
| chr7 |  | 69838308 | | 69838338 BARHL1 | |  |  |
| chr7 |  | 69851321 | | 69851351 FOXB1 | |  |  |
| chr7 |  | 69857973 | | 69858003 FERD3L | |  |  |
| chr7 |  | 69952246 | | 69952276 REST | |  |  |
| chr7 |  | 70126179 | | 70126209 BHLHE23 | |  | BHLHE23 |
| chr7 |  | 70140489 | | 70140519 BHLHE23 | |  |  |
| chr7 |  | 70188361 | | 70188391 TBR1 | |  |  |
| chr7 |  | 70208919 | | 70208949 SP9 | | SP9 | SP9 |
| chr7 |  | 70210613 | | 70210643 TBR1 | | TBR1 | TBR1 |
| chr7 |  | 70214041 | | 70214071 FOXB1 | |  |  |
| chr7 |  | 70214600 | | 70214630 REST | |  | REST |
| chr7 |  | 70219256 | | 70219286 BARHL1 | | BARHL1 | BARHL1 |
| chr7 |  | 70219588 | | 70219618 TBR1 | |  | TBR1 |
| chr7 |  | 70219609 | | 70219639 EN2 | |  |  |
| chr7 |  | 70219609 | | 70219639 GBX2 | |  |  |
| chr7 | | | 70219609 | | 70219639 GSX2 |  |  |
| chr7 | | | 70219767 | | 70219797 DUX4 |  |  |
| chr7 | | | 70220026 | | 70220056 TBR1 | TBR1 | TBR1 |
| chr7 | | | 70220026 | | 70220056 FOXB1 | FOXB1 | FOXB1 |
| chr7 | | | 70222612 | | 70222642 FOXB1 |  |  |
| chr7 | | | 70222727 | | 70222757 BARHL1 |  |  |
| chr7 | | | 70224022 | | 70224052 REST |  |  |
| chr7 | | | 70226874 | | 70226904 FERD3L |  |  |
| chr7 | | | 70228909 | | 70228939 REST |  |  |
| chr7 | | | 70251351 | | 70251381 FERD3L |  |  |
| chr7 | | | 70353421 | | 70353451 SP9 | SP9 | SP9 |
| chr7 | | | 70386005 | | 70386035 TBR1 |  |  |

***Sup.Table 2. Thirty-three TF Sites identified in the AUTS2 locus and present in modern humans as compared to archaic hominins.***

*TF sites identified using our method, TF overlap and ATACseq overlap are underlined using color lines.*

We decided to study possible correlations between AUTS2 transcripts levels and (i) these 4 transcription factors *TBR1*, *BARHL1*, *FOXB1* and *SP9*, that are validated by the three techniques, (ii) *FERD3L* and *REST*, both identified as present in ENCODE “distal enhancer like signatures” and (iii) EN2 identified as a novel binding site in our study.

In the *AUTS2* locus where we identified 33 TF sites novel in modern humans in contrast to archaic hominins, different sequences have been investigated. The first half of *AUTS2* genome sequence (human hg19; chr7:69,025,010-69,636,926) displayed the strongest statistical signal in a genomic screen differentiating modern humans from Neanderthals, suggesting an important role in human-specific evolution ^3^. This selective sweep is attributed to a stretch of 293 consecutive SNPs. Comparative genomics has identified thousands of human accelerated regions (HARs) or human accelerated conserved non-coding sequences (HACNS), that are evolutionarily conserved sequences with an unexpected number of nucleotide changes on the human lineage ^5^. Three HARs, HAR31 ^66^ in intron four and HACNS in introns one and six, HACNS 369 and HACNS 174 respectively ^67^, were identified in the *AUTS2* locus. These putative enhancers have been tested by analysis of their expression in brain in transgenic mouse embryos at E11.5 ^4^ and results deposited in the Vista Enhancer browser [(https://enhancer.lbl.gov/)](https://enhancer.lbl.gov/). The VISTA Enhancer Browser is a central resource for experimentally validated human and mouse noncoding fragments with gene enhancer activity as assessed in transgenic mice.

HACNS 369 (hs658 in Vista Enhancer Browser database) (human hg19; chr7:69,326,48769,326,735) was tested in transgenic mice. Its expression was detected in midbrain and neural tube at E11.5.

HAR31 (hs2316 in Vista Enhancer Browser database) (human hg19; chr7:69,648,01969,648,177) was tested in transgenic mice. Its expression was detected in eye at E11.5.

HACN174 (human hg19; chr7:70,017,615-70,017,729) was not tested in transgenic mice.

An ASD-associated *AUTS2* intronic deletion of 33,519 bp (human hg19; chr7:69,699,07469,732,592) was identified in intron 4 ^68^. Three enhancer sequences have been tested in transgenic mice with their expression pattern reported at E11.5 in the Vista Enhancer Browser database and in ^69^: hs658 is expressed in midbrain, medulla and neural tube; hs2317 is expressed in eye and olfactory epithelium; hs2318 is expressed in midbrain, hindbrain, forebrain and cerebellum anlagen at E.11.5.

We analyzed if novel sites present in modern humans and not in archaic hominins were present in these three types of regions. Interestingly, no novel TF binding sites were present (**Sup. Fig. 7**). It is interesting to note that only 8% of HAR substitutions are not observed in the archaic hominins, indicating that 92% of HARs are related to modern humans-chimpanzee divergence that occurred at ~6My (**Sup. Fig. 1**) ^70^.

***Identification of subtypes of neurons and neuronal networks where AUTS2 levels correlate with novel transcription factors found in modern humans as compared to archaic hominins***

We first used adult brain (prefrontal cortex and anterior cingular cortex) single-cell transcriptome database from ^71^ displayed at [https://genome.ucsc.edu.](https://genome.ucsc.edu/) We identified that AUTS2 is expressed in both excitatory and inhibitory neurons and in a variety of non-neuronal brain cell types (**Sup. Fig. 11A**).

We analyzed transcription factors which have novel sites in *AUTS2* locus in modern humans as compared to archaic hominins. TBR1 is specifically expressed in both excitatory and inhibitory neurons but not in non-neuronal brain cell types (**Sup. Fig. 11B**). FOXB1 is expressed in both excitatory and inhibitory neurons but also in all non-neuronal brain cell types (**Sup. Fig. 11C**). FERD3L is expressed in excitatory cortex in layers 2/3 and 4 and neuron NRGN +I subtype (**Sup. Fig. 12A**). REST is specifically expressed in astrocytes, microglia, oligodendrocyte precursors and oligodendrocytes (Sup. Fig. 12B). SP9 is specifically expressed in interneurons (PVALB+, SST+, SV2C+, VIP+ subtypes) (**Sup. Fig. 12C**). We also found that *EN2* and *BARHL1* are specifically expressed in adult human cerebellum, out of the 52 tissues and 2 cell lines studied (**Sup. Fig. 13**).


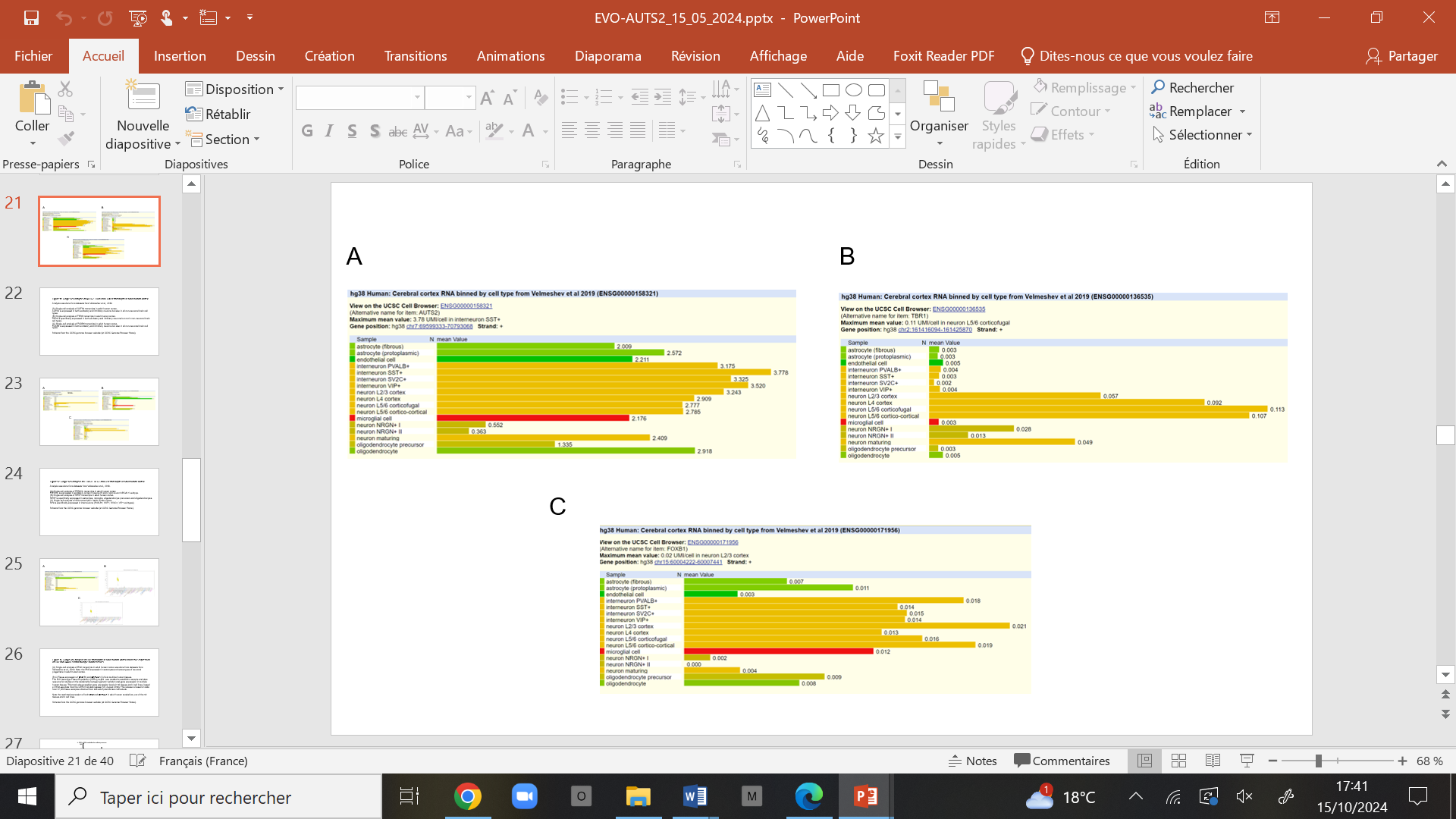


***Supplemental figure 11. Single-cell analysis of AUTS2, TBR1 and FOXB1 transcripts in adult human cortex.***

*Analysis was done from datasets from Velmeshev et al., 2019.*

*(A) Single-cell analysis of AUTS2 transcripts in adult human cortex.*

*AUTS2 is expressed in both excitatory and inhibitory neurons but also in all non-neuronal brain cell types.*

*(B) Single-cell analysis of TBR1 transcripts in adult human cortex.*

*TBR1 is specifically expressed in both excitatory and inhibitory neurons but not in non-neuronal brain cell types.*

*(C) Single-cell analysis of FOXB1 transcripts in adult human cortex.*

*FOXB1 is expressed in both excitatory and inhibitory neurons but also in all non-neuronal brain cell types.*

*Schema from the UCSC genome browser website (at UCSC Genome Browser Home).*


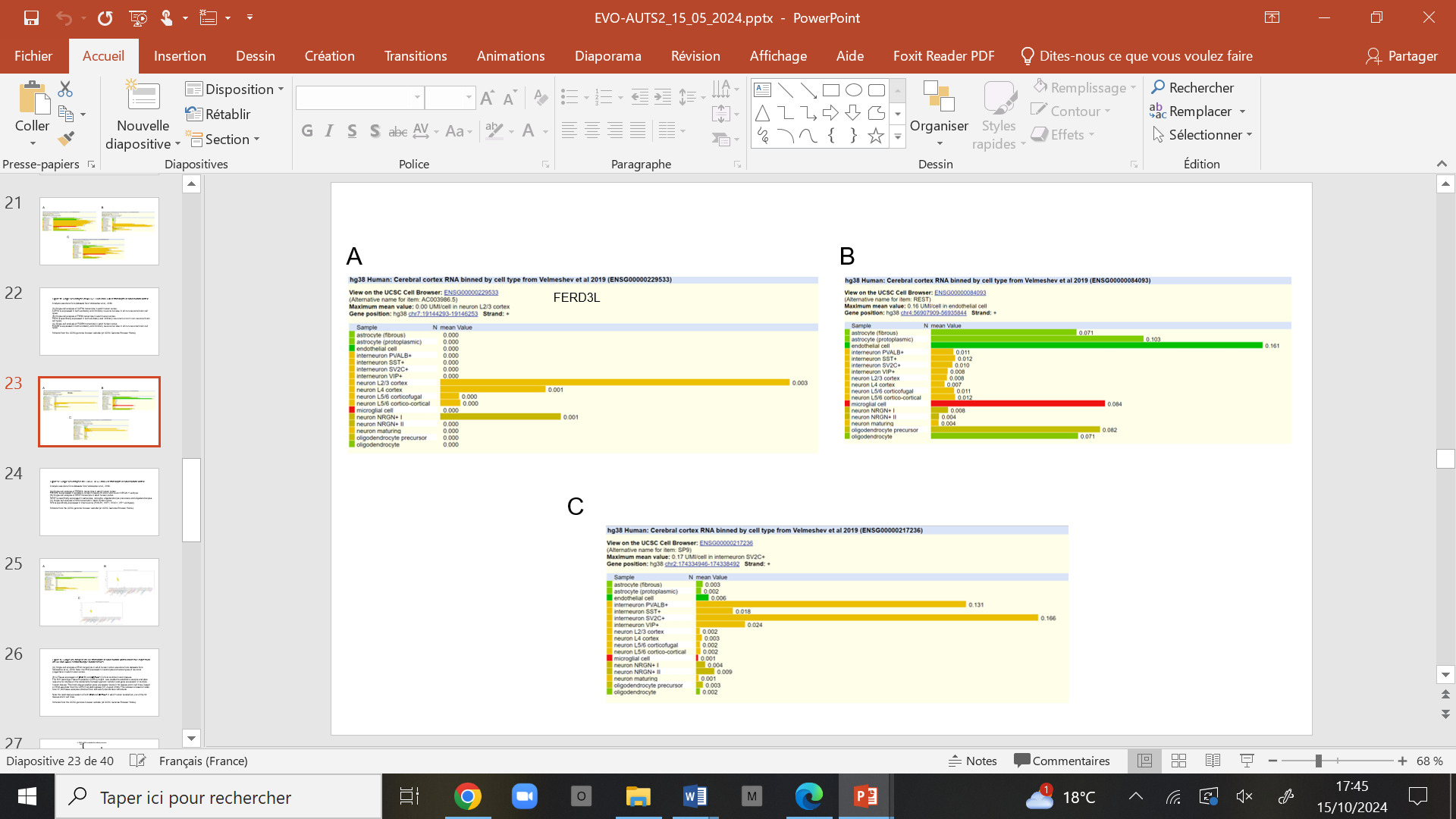


***Supplemental figure 12. Single-cell analysis of FERD3L, REST and SP9 transcripts in adult human cortex.***

*Analysis was done from datasets from Velmeshev et al., 2019.*

*(A) Single-cell analysis of FERD3L transcripts in adult human cortex.*

*FERD3L is expressed in excitatory cortex in layers 2/3 and 4 and neuron NRGN +I subtype.*

*(B) Single-cell analysis of REST transcripts in adult human cortex.*

*REST is specifically expressed in astrocytes, microglia, oligodendrocyte precursors and oligodendrocytes.*

*(C) Single-cell analysis of SP9 transcripts in adult human cortex.*

*SP9 is specifically expressed in interneurons (PVALB+, SST+, SV2C+, VIP+ subtypes) Schema from the UCSC genome browser website (at UCSC Genome Browser Home).*


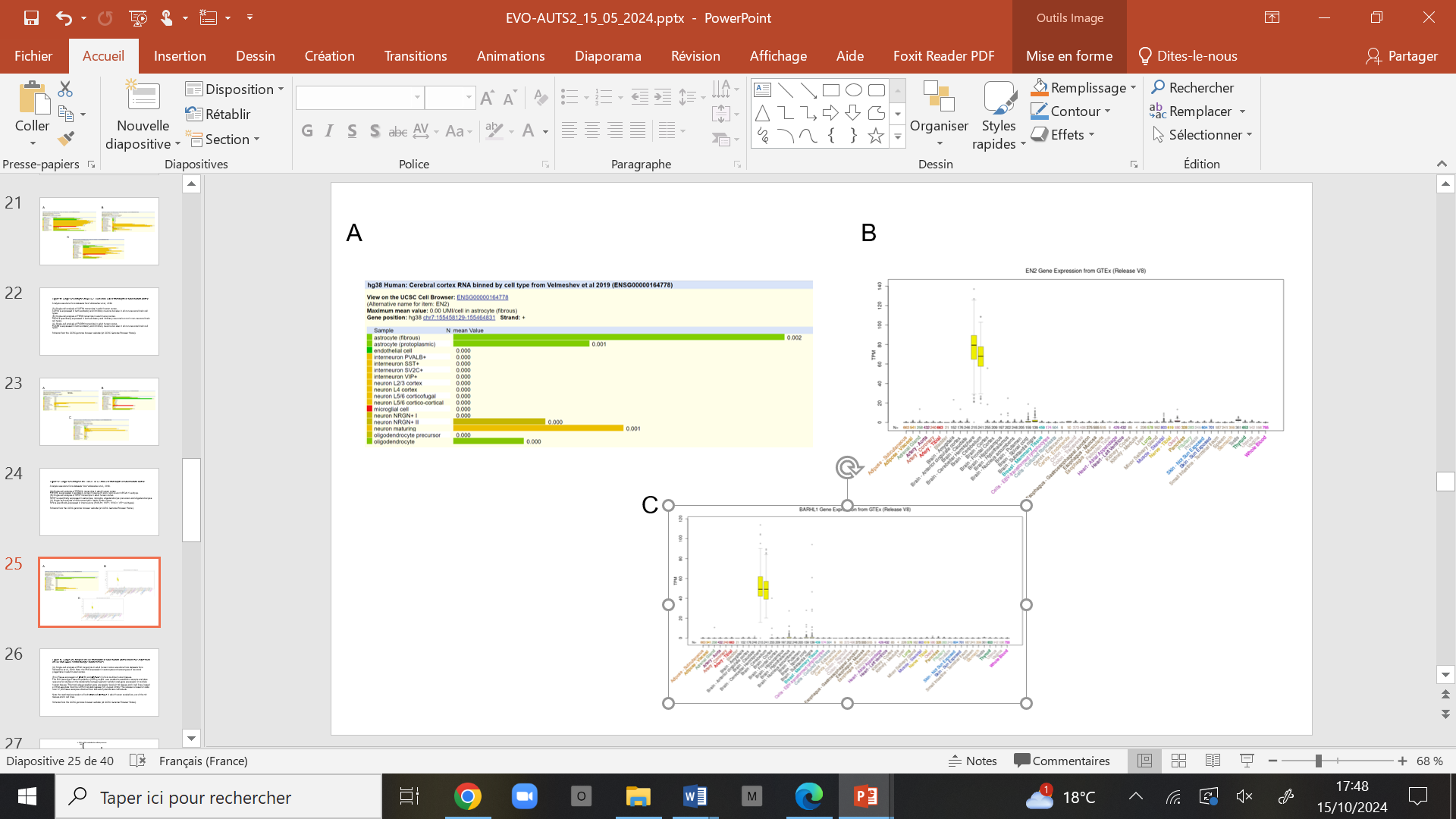


***Supplemental figure 13.*** ***Single-cell analysis of EN2 transcripts in adult human cortex and tissue expression of EN2 and BARHL1 from multiple human tissues.***

*(A) Single-cell analysis of EN2 transcripts in adult human cortex was done from datasets from Velmeshev et al., 2019. Note the EN2 expression in astrocytes and some types of neuronal progenitors in adult human cortex.*

*(B, C) Tissue expression of EN2 (B) and BARHL1 (C) from multiple human tissues.*

*The NIH Genotype-Tissue Expression (GTEx) project was created to establish a sample and data resource for studies on the relationship between genetic variation and gene expression in multiple human tissues. This track shows median gene expression levels in 52 tissues and 2 cell lines, based on RNA-seq data from the GTEx final data release (V8, August 2019). This release is based on data from 17,382 tissue samples obtained from 948 adult post-mortem individuals.*

*Note the restricted expression of both EN2 and BARHL1 in adult human cerebellum, out of the 52 tissues and 2 cell lines.*

We evidenced significant TBR1-AUTS2 correlation in excitatory neurons (**Sup. Fig. 14A**) and FOXB1-AUTS2 correlation in inhibitory and excitatory neurons (**Sup. Fig. 14B**).


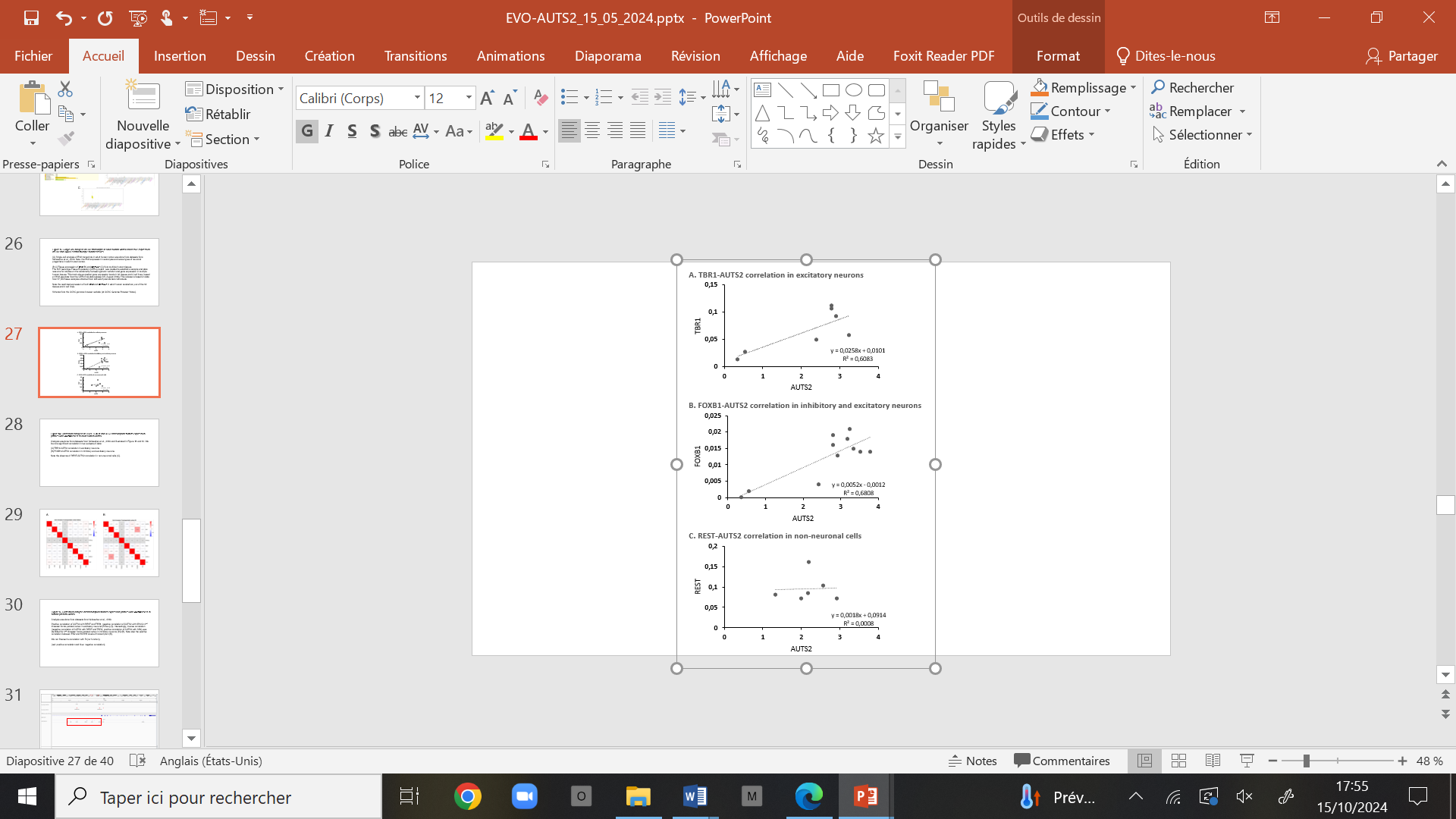


***Supplemental figure 14. Correlation analysis of TBR1, FOXB1 and REST transcription factors expression profiles with AUTS2 levels in adult human cortex.***

*Analysis was done from datasets from Velmeshev et al., 2019 and illustrated in Figure 11 and 12. We found a significant correlation in two subsets of data:*

*(A)TBR1-AUTS2 correlation in excitatory neurons.*

*(B)FOXB1-AUTS2 correlation in inhibitory and excitatory neurons.*

*Note the absence of REST-AUTS2 correlation in non-neuronal cells (C).*

We next took advantage of a recent human brain single-cell database that covers prenatal to adult stages ^72^. This recent study analyzed >700,000 single-nucleus RNA sequencing profiles of human brain from 106 donors during prenatal and postnatal developmental. Multiple time points were studied: human prenatal (two fetal stages: 2^nd^ trimester, 3^rd^ trimester), early postnatal (0-1 years, 1-2 years, 2-4 years) and late postnatal-adult (4-10 years, 10-20 years, adult) for eight main lineages and cell types (microglia, astrocytes, vascular cells, glial progenitors, OPCs, interneurons, excitatory neurons and oligodendrocytes). Furthermore, ten regions were examined: prefrontal, cingulate, temporal, Insula, motor cortex and whole cortex, medial ganglionic eminence (MGE), lateral ganglionic eminence (LGE), caudal ganglionic eminence (CGE) and ganglionic eminence (GE) to reconstruct interneurons lineages. From these datasets, we were able to characterize 386 distinct subsets of data to study possible correlation between AUTS2 transcripts and seven transcription factors that display increase in numbers for *AUTS2* locus during modern human evolution. From these 386 subsets, we characterized only 22 subsets that display statistically significant correlation between AUTS2 transcript levels and levels of REST, SP9 and TBR1 (**Sup. Table 3**). Interestingly, these subsets are related to 2^nd^ trimester (n=8), 3^rd^ trimester of gestation (n=8) and the 0-1 years postnatal period (n=6). Correlation involved both excitatory and inhibitory neurons in fronto-parietal cortex and BA8 cortical region (n=6) both also non-neuronal cells (n=16).

| **REST SP9** | | | **TBR1**  3,008E-10 |
| --- | --- | --- | --- |
| 0-1 years BA8 ExNeu | 2,07595E-32 | 8,89E-17 |  |
| 0-1 years BA8 IN | 2,07595E-32 | 8,89144E-17 | 3,008E-10 |
| 0-1 years BA8 AST | 2,07595E-32 | 8,89144E-17 | 3,008E-10 |
| 0-1 years BA8 MG | 2,07595E-32 | 8,89144E-17 | 3,008E-10 |
| 0-1 years BA8 VASC | 2,07595E-32 | 8,89144E-17 | 3,008E-10 |
| 0-1 years BA8 OPC | 2,07595E-32 | 8,89144E-17 | 3,008E-10 |
| 3rd trimester Frontoparietal cortex ExNeu | 2,9665E-161 | 3,406E-118 | 1,548E-16 |
| 3rd trimester Frontoparietal cortex AST | 2,9665E-161 | 3,406E-118 | 1,548E-16 |
| 3rd trimester Frontoparietal cortex MG | 2,9665E-161 | 3,406E-118 | 1,548E-16 |
| 3rd trimester Frontoparietal cortex OPC | 2,9665E-161 | 3,406E-118 | 1,548E-16 |
| 3rd trimester Frontoparietal cortex IN | 2,9665E-161 | 3,406E-118 | 1,548E-16 |
| 3rd trimester Frontoparietal cortex VASC | 2,9665E-161 | 3,406E-118 | 1,548E-16 |
| 3rd trimester Frontoparietal cortex GLIALPROG | 2,9665E-161 | 3,406E-118 | 1,548E-16 |
| 3rd trimester Frontoparietal cortex OL | 2,9665E-161 | 3,406E-118 | 1,548E-16 |
| 2nd trimester Frontoparietal cortex ExNeu | 4,53286E-54 | 3,07569E-91 | 3,004E-10 |
| 2nd trimester Frontoparietal cortex GLIALPROG | 4,53286E-54 | 3,07569E-91 | 3,004E-10 |
| 2nd trimester Frontoparietal cortex IN | 4,53286E-54 | 3,07569E-91 | 3,004E-10 |
| 2nd trimester Frontoparietal cortex AST | 4,53286E-54 | 3,07569E-91 | 3,004E-10 |
| 2nd trimester Frontoparietal cortex VASC | 4,53286E-54 | 3,07569E-91 | 3,004E-10 |
| 2nd trimester Frontoparietal cortex MG | 4,53286E-54 | 3,07569E-91 | 3,004E-10 |
| 2nd trimester Frontoparietal cortex OPC | 4,53286E-54 | 3,07569E-91 | 3,004E-10 |
| 2nd trimester Frontoparietal cortex OL | 4,53286E-54 | 3,07569E-91 | 3,004E-10 |

***Sup.Table 3. Correlation of expression of AUTS2 with three TFs whose novel sites were detected in modern humans as compared to archaic hominins.***

*(BA8: Brodmann Area 8, ExNeu: excitatory neurons; IN: interneurons; AST: astrocytes; MG: microglia; VASC: vascular cells; OPC: oligodendrocyte precursor cell; OL: oligodendrocyte)*


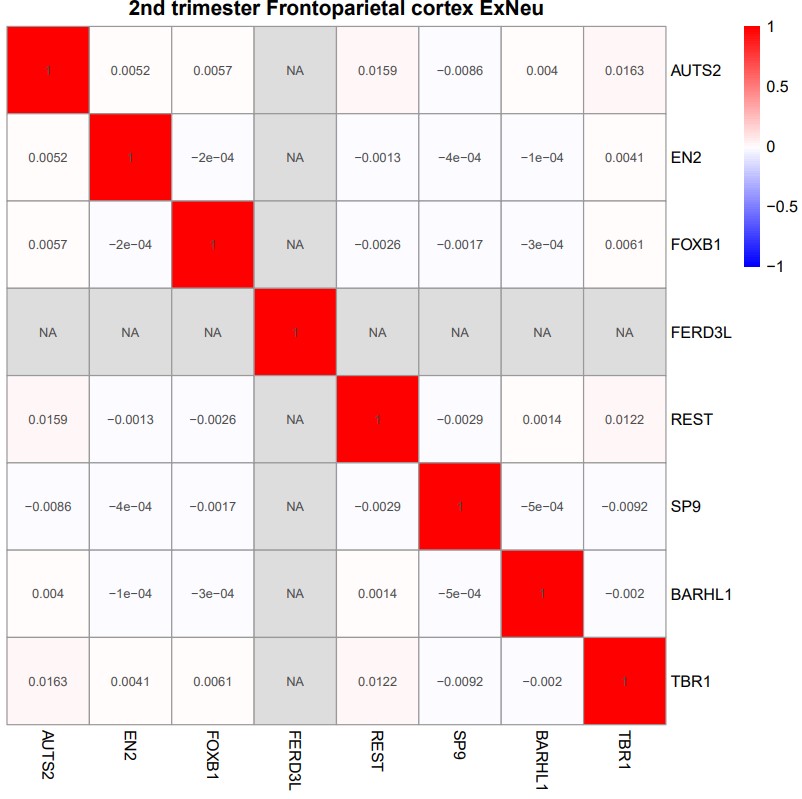

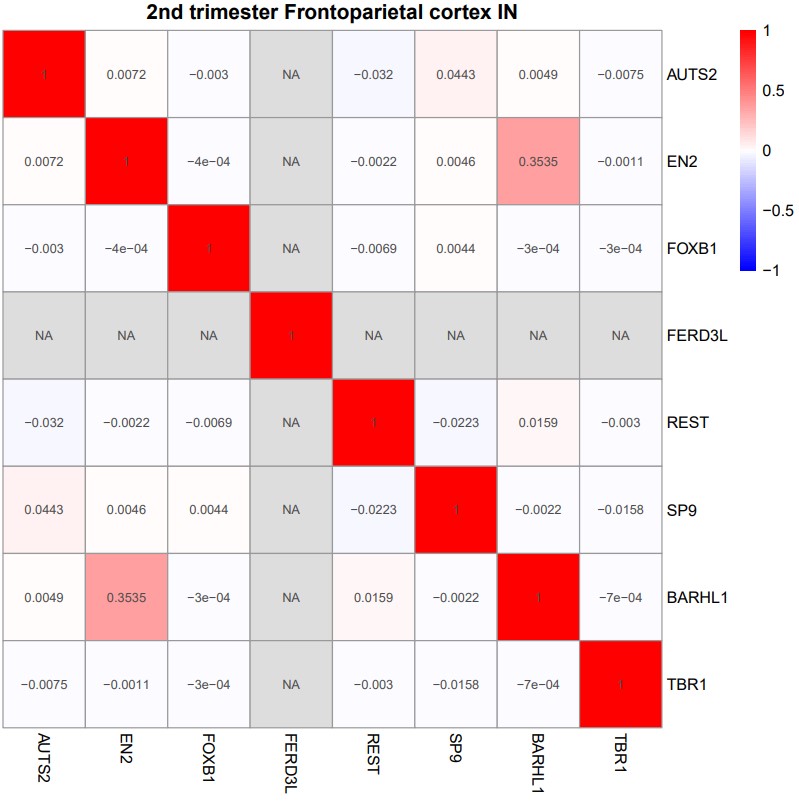


***Supplemental figure 15. Correlation analysis of transcription factors expression profiles with AUTS2 levels in human prenatal cortex.***

*Analysis was done from datasets from Velmeshev et al., 2019.*

*Positive correlation of AUTS2 with REST and TBR1, negative correlation of AUTS2 with SP9 for 2nd trimester fronto-parietal cortex in excitatory neurons (Exneu) (A). Interestingly, inverse correlation (negative correlation of AUTS2 with REST and TBR1, positive correlation of AUTS2 with SP9) was identified for 2nd trimester fronto-parietal cortex in inhibitory neurons (IN) (B). Note also the positive correlation between EN2 and BARH1 levels of transcription (B).*

*We ran Pearson’s correlation with R (cor function).*

*(red: positive correlation and blue: negative correlation).*

We illustrate two examples of correlation of AUTS2 levels with expression of three TFs, SP9, TBR1 and REST out of seven TFs studied (**Sup. Fig. 15**). We found positive correlation of AUTS2 with REST and TBR1, negative correlation of AUTS2 with SP9 for 2^nd^ trimester fronto-parietal cortex in excitatory neurons (Exneu) (**Sup. Fig. 15A**). Interestingly, inverse correlation (negative correlation of AUTS2 with REST and TBR1, positive correlation of AUTS2 with SP9) was identified in interneurons (IN) (**Sup. Fig. 15B**). Also note the positive correlation between EN2 and BARH1 levels of transcription (**Sup. Fig. 15C**).

Altogether, these results demonstrate that SP9, TBR1 and REST TFs have a concerted action on AUTS2 expression in distinct types of cortex cells, including both excitatory neurons and interneurons.

***Identification of an AUTS2 sequence able to reveal functional impacts of AUTS2 transcriptional changes in modern humans as compared to archaic hominins***


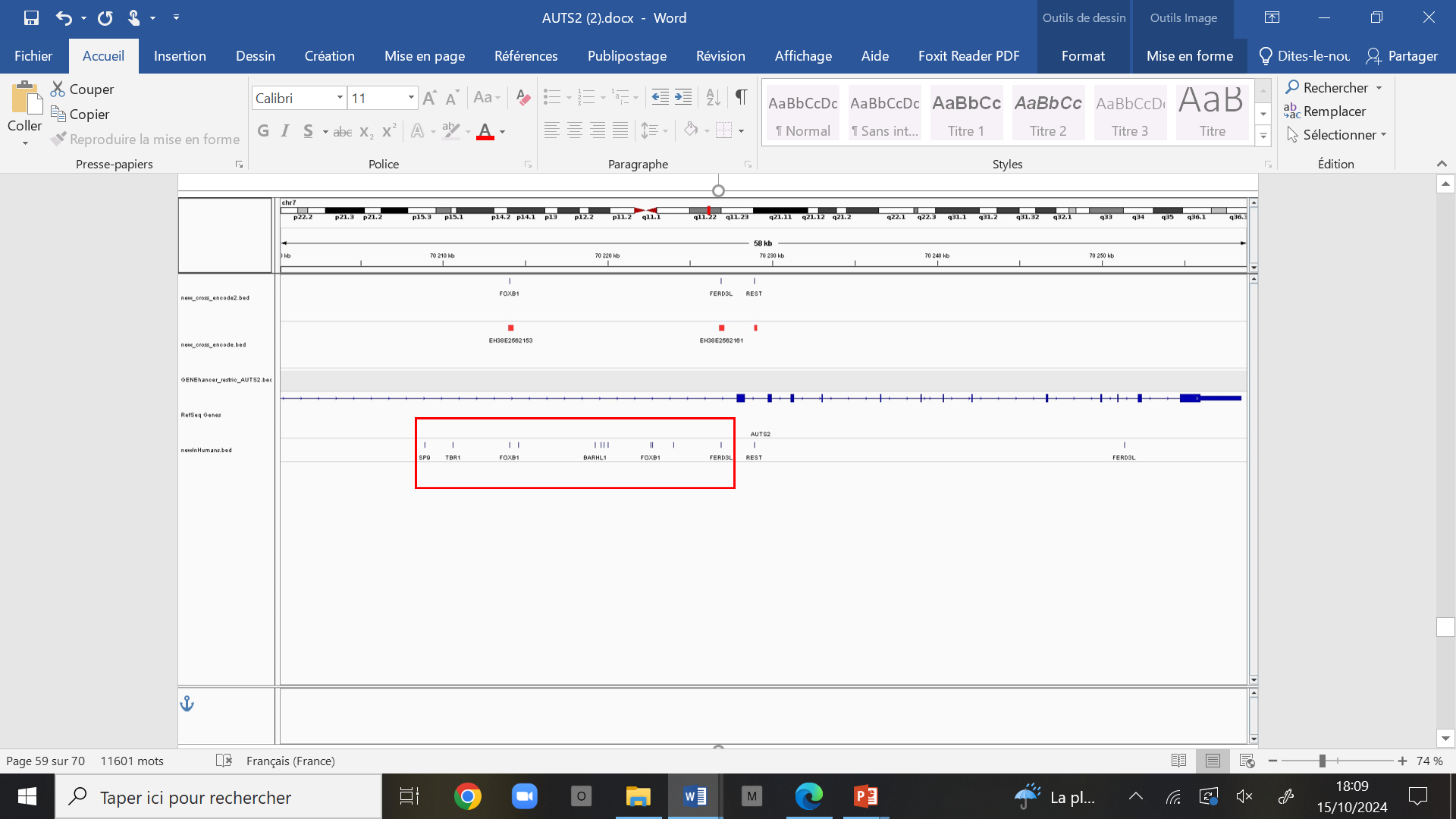


***Supplemental figure 1­6. An AUTS2 sequence able to reveal functional impacts of AUTS2 transcriptional changes in modern humans as compared to archaic hominins***

*We identified a ~20 kb genomic region (red-boxed) located in intron 7 of AUTS2 that includes 16 TFs binding sites novel in modern humans as compared to archaic hominins.*

*This region starts with a SP9 site (chr7:70,208,919) and ends with a FERD3L site (chr7: 70,226,904), from hg19 data. The 16 TFs binding sites include SP9, TBR1, BARHL1, FOXB1 sites found in the three distinct analyses used.*

*Two enhancers classified as ENCODE “distal enhancer-like signature”: EH38E2562153*

*(chr7:70,213,974-70,214,299) with a FOXB1 site and EH38E2562161 (chr7:70,226,783-70,227,081) with a FERD3L site.*

From the results presented here, we can identify a sequence of ~20kb of genomic sequence from the intron seven of *AUTS2* gene (**Sup. Fig. 16**). This sequence includes 16 TFs out of 33 TFs binding sites novel in modern humans as compared to archaic hominins and identified in the *AUTS2* locus. This region starts with a SP9 site (chr7:70,208,919) and ends with a FERD3L site (chr7: 70,226,904), from hg19 data. The 16 TFs binding sites include SP9, TBR1, BARHL1, FOXB1 sites found in the three distinct analyses used. Furthermore, this ~20kb region includes two enhancers classified as ENCODE “distal enhancer-like signature”. EH38E2562153 (chr7:70,213,974-70,214,299) includes a FOXB1 site and EH38E2562161 (chr7:70,226,783-70,227,081) a FERD3L site.

Interestingly, we have an EN2, BARHL1 and FERD3L/NATO3 sites in this sequence. EN2 and BARHL1 are expressed in the same type of neurons in cerebellum (**Sup. Fig. 17**). Furthermore, both EN2 and FERD3L/NATO3 are expressed in developing dopaminergic neuron precursors of the midbrain ^60,73,74^.

Interestingly, in relation with this ~20kb region of exon 7, one can note that the AUTS2 syndrome severity is worse when mutations involve 3’ regions of the *AUTS2* gene as compared to mutations of 5’ region ^6^.


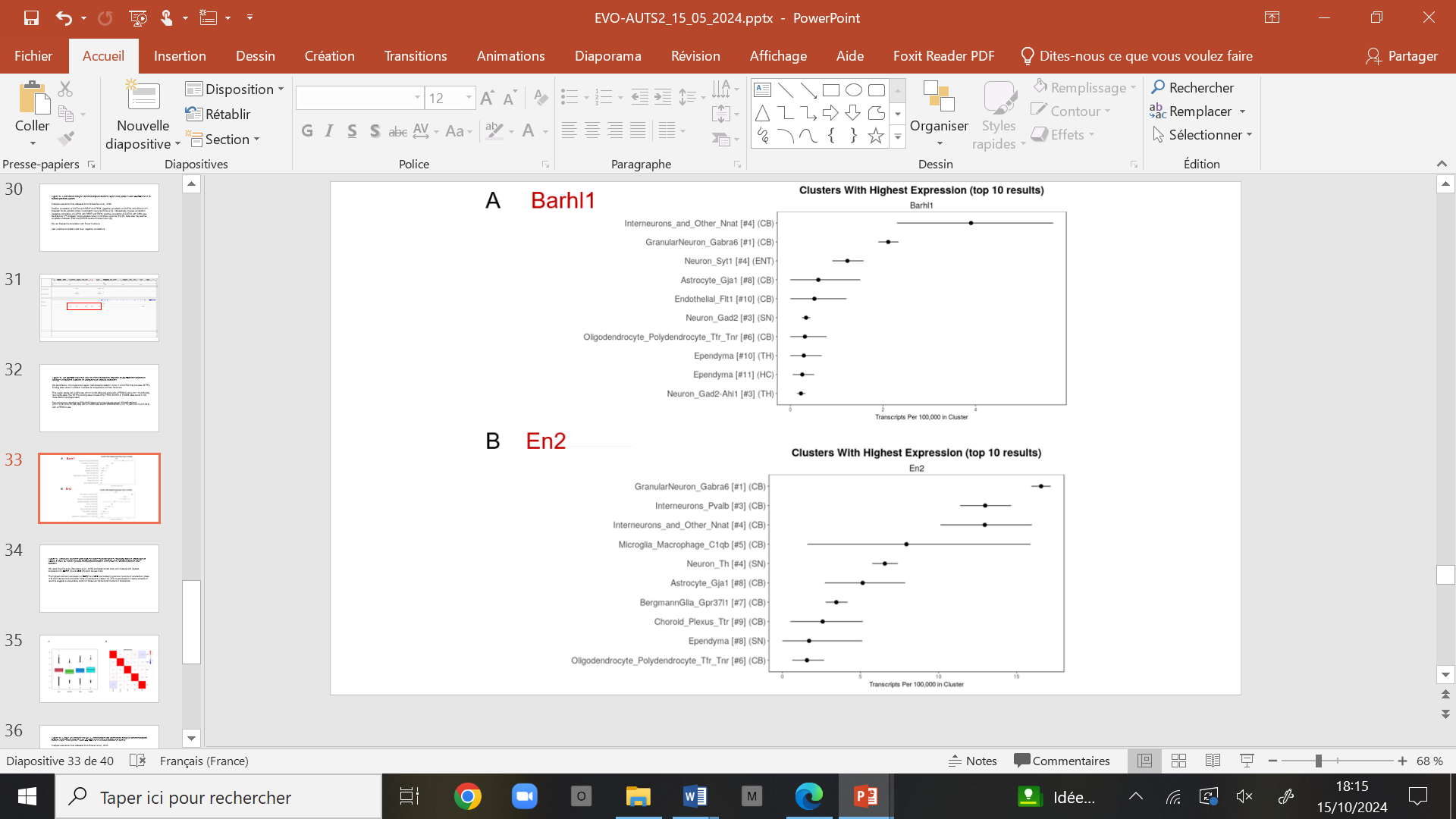


***Supplemental figure 1­7. Brain cell clusters with highest expression for genes encoding mouse orthologs of BARHL1 and EN2 brain-specific transcription factors lost between extinct hominins and humans.***

*We used DropViz suite (Saunders et al., 2018) and determined brain-cell clusters with highest expression for Barhl1 (A) and En2 (B) adult mouse brain.*

*The highest common expression of Barhl1 and En2 are located in granular neurons of cerebellum (class n°1) and interneurons and other Nnat of cerebellum (class n°4). This co-expression in same subsets of neurons suggest a cooperative action of these two transcription factors in cerebellum.*

***Correlation of AUTS2 levels with transcription factors in marmoset and mouse brains***

We studied single-cell analysis of AUTS2 transcripts and correlation analysis of transcription factors expression profiles with *AUTS2* levels in marmoset and mouse cortex, both at adult and prenatal stages. Only adult data were available for marmoset ^75^ (**Sup. Fig. 18**). In adult cortex of marmoset, AUTS2 is expressed in both excitatory and inhibitory neurons but also in astrocytes and oligodendrocytes (**Sup. Fig. 18A**). From the marmoset adult brain single-cell data, we found only a correlation between *AUTS2* and *TBR1* transcript levels for glutamatergic neurons (Pval = 4.98 e-27) (**Sup. Fig. 18B**).


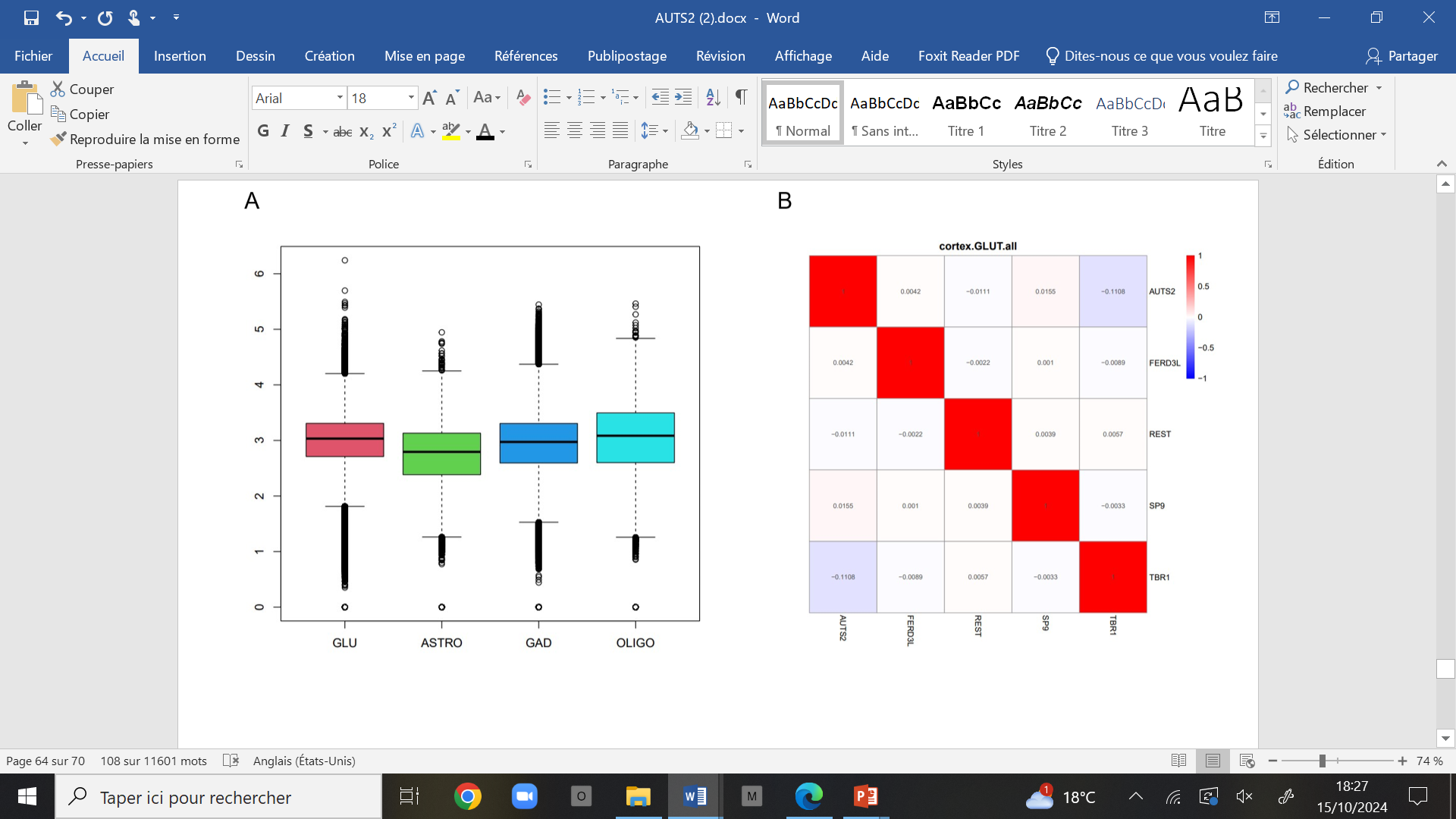


***Supplemental figure 1­8.*** ***Supplemental figure 18. Single-cell analysis of AUTS2 transcripts and correlation analysis of transcription factors expression profiles with AUTS2 levels in adult marmoset cortex.***

*Analysis was done from datasets from Krienen et al., 2023.*

*(A) Boxplots of AUTS2 detected per cell in cortex. Note the AUTS2 expression in excitatory neurons (GLU), astrocytes (ASTRO), interneurons (GAD) and oligodendrocytes (OLIGO).*

*(B) Correlation analysis of FERD3L, REST, SP9 and TBR1 transcription factors expression profiles with AUTS2 levels, for glutamatergic neurons.*

*We ran Pearson’s correlation with R (cor function).*

*Note a positive correlation between SP9 and AUTS2 levels and negative correlation between TBR1 and AUTS2 levels.*

*(red: positive correlation and blue: negative correlation).*


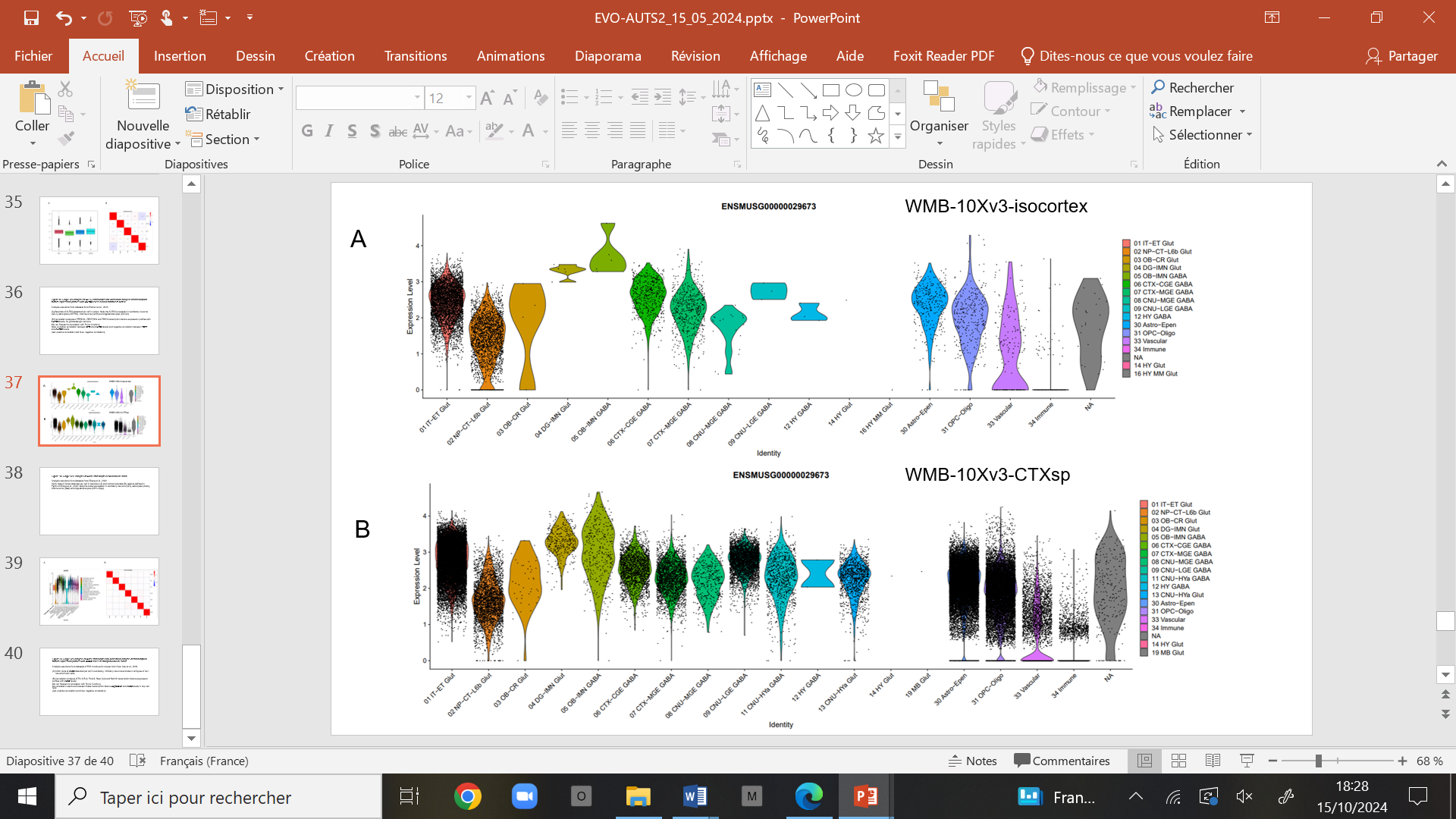


***Supplemental figure 1­9.*** ***Single-cell analysis of Auts2 transcripts in adult mouse brain.***

*Analysis was done from datasets from Zhang et al., 2023.*

*Violin plots of Auts2 detected per cell in isocortex (A) and cortical subplate (B) regions defined in Fig1C of Zhang et al., 2023. Note the Auts2 expression in excitatory neurons (Glu), astrocytes (Astro), interneurons (Gad) and oligodendrocytes (OPC-Oligo).*

We next analyzed single-cell transcriptomics for adult mouse brain using data set from ^76^ (Sup. Fig. 19). We used single-cell data for isocortex (WMB-10Xv3-Isocortex) (Sup. Fig. 19A) and cortical subplate (WMB-10Xv3-CTXsp) (Sup. Fig. 19B) regions defined in Fig1C of ^76^. Cortical subplate region was characterized as in the Allen brain atlas ([https://mouse.brainmap.org/static/atlas)](https://mouse.brain-map.org/static/atlas) and includes claustrum, endopiriform nucleus and amygdala nuclei. As for human and marmoset, *AUTS2* is expressed in both excitatory and inhibitory neurons but also in astrocytes and oligodendrocytes for both isocortex (Sup; Fig. 19A) and CTXsp regions (Sup. Fig. 19B). We next examined correlation of Auts2 with the seven transcription factors whose binding sites increased in modern humans as compared to archaic hominins (Tbr1, En2, FoxB1, Rest, Ferd3l, Sp9, Barhl1). For the neocortical set no correlation was found. For the cortical subplate set (WMB-10Xv3-CTXsp), only a correlation between Auts2 and Rest was found in inhibitory neurons (multi testing pval = 0.00059). Correlation between Auts2 and Rest is in agreement with recent data that suggest that Rest knockout increases excitability in neural networks. For prenatal mouse brain, we used the data from the Mouse Organogenesis Cell Atlas (MOCA) for mouse E13.5 brain ^79^. *Auts2* was detected per cell in excitatory, inhibitory neurons and also in all types of non-neuronal brain cells (Sup. Fig. 20A). We studied correlation of Tbr1, En2, Foxb1, Rest, Sp9 and Barhl1 transcription factors expression profiles with *Auts2* levels. No correlation was found between these transcription factors *expression* and *Auts2* levels in any cell type (Sup. Fig. 20B).

Altogether, correlations found in marmoset and mouse are extremely more restricted than those found in humans, suggesting an evolutionary mechanism in the selection of TFs sites during mammalian lineage.


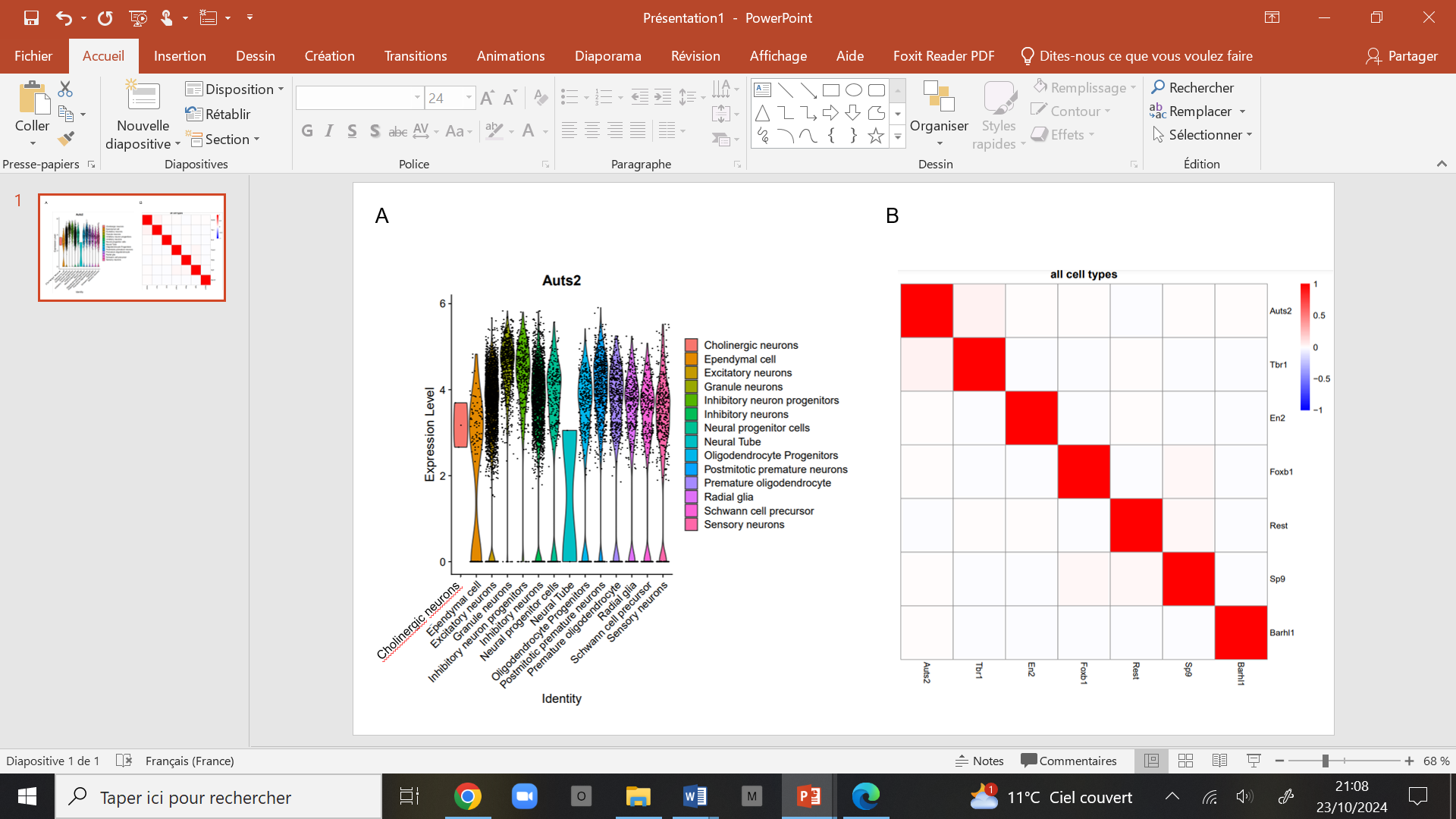


***Supplemental figure 20. Single-cell analysis of Auts2 transcripts and correlation analysis of transcription factors expression profiles with Auts2 levels in embryonic mouse brain.***

*Analysis was done from datasets of E13.5 embryonic mouse brain from Cao et al., 2019.*

*(A)Violin plots of Auts2 detected per cell in excitatory, inhibitory neurons and also in all types of non-neuronal brain cells.*

*(B) Correlation analysis of Tbr1, En2, Foxb1, Rest, Sp9 and Barhl1 transcription factors expression profiles with Auts2 levels.*

*We ran Pearson’s correlation with R (cor function).*

*No correlation was found between these transcription factors expression and Auts2 levels in any cell type.*

*(red: positive correlation and blue: negative correlation).*

***Prediction of neural networks involved in autism spectrum disorders***

Recent identification of risk genes for psychiatric disorders have set the stage for functional interrogation of disease related-circuits and underlying mechanisms of pathophysiology ^80^. Here, we propose that *AUTS2* expression within mammalian lineage can be a predictor of neural networks involved in ASD. Our working hypothesis is that the distribution of *AUTS2* transcripts in different mammalian models can be instrumental to predict behavioral phenotypes.

Behavioral studies have been conducted using several different types of *Auts2* mutant mice ^2,22,45,81–83^.

In the first study, across early development, knockout (KO) mice were deficient in ultrasonic vocalizations (USVs) emitted ^2^. In the study of Hori et al., 2015, *Auts2* heterozygotic mutant mice displayed behavioral abnormalities in anxiety-related emotions (elevated plus maze; cued fear associative memory but no changes in contextual fear associative memory) and recognition memory abnormalities ^81^.

Three studies were based on conditional KO models leading to KO of *Auts2* in excitatory neurons of the forebrain ^82^, in the cerebellum ^22^ or in developing forebrain ^83^. Selective deletion of *Auts2* in excitatory neurons in the adult forebrain ^82^ induces a phenotype different from the constitutive KO ^81^. In this conditional KO, KO mice display social deficits and altered vocal communication. Interestingly, the number of USVs but also the complexity of USV syllables emitted from male mice during courtship behaviors are reduced ^82^. Selective deletion of *Auts2* in the cerebellum induces behavioral impairments in motor learning and vocal communications ^22^. Selective deletion of *Auts2* in developing forebrain led to hypoplasia of DG with social deficits and stereotypies (excessive grooming and digging behaviors) ^83^.

The third type of *Auts2* mouse model developed by our group ^45^ is based on either deletion or duplication of the ~1.2 Mb *Auts2* locus. We evidenced repetitive and restricted behaviors (rearing) in Del/+ mice.

Auts2 gene expression was found increased by repeated cocaine administration specifically in D_2_-type medium spiny neurons in the nucleus accumbens ^84^.

Altogether, these results indicate that Auts2 is involved in cognition and recognition memory, social memory, stereotypies and perseverative behaviors, anxiety and addiction.

Cognition is related with Auts2 expression in Frontal Cortex and Hippocampus. Defects in Auts2 in these regions can be linked to ID found in patients.

Recognition memory involves Hippocampus and related structures such as Lateral Entorhinal Cortex that express AUTS2 in developing marmoset brain. Recognition memory circuitry is linked to dyslexia ^27,28^. AUTS2 locus was recently found associated with dyslexia ^16^. Social brain involves Frontal Cortex, Temporal Cortex and Amygdala ^85^ but also Hippocampus and Hypothalamus ^23^. Deregulation of Aust2 expression in these regions can induces abnormal social interactions and altered vocal communication as found in the *Auts2* mouse models. Stereotypes and perseverative behaviors (excessive grooming and digging behaviors; rearing) are linked to frontal cortex-striatum-thalamus loops that express *Auts2* in mouse and marmoset. Anxiety changes were evidenced in different models suggesting an expression of *Auts2* in Amygdala. This expression is well documented in neonate marmoset.

AUTS2 expression is found in claustrum, in neonate marmoset. This region is linked to high level cognition ^32^. Interestingly, each single claustrum neuron in mouse brain displays widespread extensive connectivity with the entire cerebral cortex ^34^.

Further work is needed to demonstrate if Aust2 is expressed in claustrum neurons in mouse. We also found *AUTS2* expression in GE in human fetal brain. Subcortical structures such as Striatum, Pallidum and Amygdala are derived from progenitors that originate in GE ^40,86^. GE generate interneurons whose number and diversity increase in primates compared to rodents ^40,86^. It would be important to identify if mouse GE and/or interneurons derived from GE express Auts2 and to manipulate their level of Auts2 expression.

One of the questions raised by the analysis of open dataset publicly available is their diversity in terms of timepoints, especially for prenatal stages. For instance, Genepaint [(https://gp3.mpg.de/)](https://gp3.mpg.de/) documents only mouse E14.5 brain data when VISTA enhancer browser [(https://enhancer.lbl.gov/)](https://enhancer.lbl.gov/) gives information on mouse E11.5 embryo. Furthermore, Marmoset Gene Atlas data [(https://gene-atlas.brainminds.jp/)](https://gene-atlas.brainminds.jp/) generated by Riken institute are restricted to neonate, 1 month, 3 months,6 months and above one year.

In the near future, large scale dataset of spatial transcriptomics will be available to complete the analysis. Spatial transcriptomics (ST) technologies provide new tools to identify the cellular organization and interactions of brain cells. Multiple ST technologies have been developed and applied to mouse and human brains. However, sequencing-based ST technologies such as the 10x Genomics Visium platform and Slide-seq ^87^, are based on a spot-by-gene matrix structure and require additional techniques to obtain the cellular information. Commercial MERFISH ^88^ and 10x Genomics Xenium ^89^ allows a subcellular resolution but are limited by their gene throughput, with hundreds of customized genes detected. In their recent study, Yao et al ^90^ reported MERFISH data on mouse brain spatial transcriptomics but Aust2 is not present in the 500 probes used.

Analysis of *AUTS2* locus sequences from extinct hominins and Humans suggest that Human *AUTS2* gene may display a distinct spatial and temporal pattern neuronal regulation of its expression when compared to extinct hominins *AUTS2* locus. Moreover, the observed differences in transcription factor sites at the *AUTS2* locus may signify a novel expression pattern of the human as compared to extinct hominins *AUTS2* locus, raising the possibility that these changes may have contributed to the brain evolution during the ~804,000 years separating extinct hominins and Human lineages. Taking in account the *AUTS2* expression in cerebellum ^22^, it would of great interest to analyze, in transgenic mouse, the potential function of the new EN2 site that we evidenced in humans but not in Neanderthals and Denisovans.

In this study, we were able to identify a ~20kb *AUTS2* sequence able to reveal functional impacts of AUTS2 transcriptional changes in modern humans as compared to archaic hominins. This sequence includes 16 TFs out of 33 TFs binding sites novel in modern humans as compared to archaic hominins. Genomic engineering strategies are now routinely used to test human sequences in mouse genome ^91^. The modern human or the archaic hominin sequences can be inserted in intron 7 of Auts2 mouse genome by CRISPR/Cas9 and homologous recombination as in ^91^. Comparison of brain development, effect on local circuits and behavior may provide novel insights on the pathophysiology of ASDs.

Altogether, our data can permit to design future studies to unravel pathophysiological phenotypes linked to AUTS2 abnormal expression.

**Detailed Materials and Methods**

**Human sample preparation**

Tissues were obtained from spontaneously or voluntarily terminated pregnancies following the informed consent of the parents and according to the French Ethical Committee recommendations. We studied embryos of 8 weeks and fetuses of 15 and 19 weeks. Tissues were fixed in 4% paraformaldehyde, embedded in paraffin and sectioned (5 μm).

**Probe synthesis and in situ hybridization**

We used human cDNA clones from the RZPD Library (ID DKFZp547C245Q2; ID IRALp962D1923Q2). We synthesized ^35^S-labeled riboprobes for Nogo (700 bp riboprobe) and NgR (1200 bp), using the P1460 riboprobe in vitro transcription system (Promega). Hybridization was carried out as previously described ^20^. Hybridizations were performed with both antisense and sense riboprobes. No signal was obtained with sense riboprobes. Expression was quantified with a Biospace Micro Imager and Betavision analysis software (Biospace Instruments) ^37^. Additional adjacent sections were stained with hematoxylin/eosin/safranin (HES) for histological examination.

**Selection of sites:**

We downloaded VCF files for Denisovan hominins, Neanderthals and ancient humans from [http://cdna.eva.mpg.de/neandertal/Vindija/VCF/Denisova/.](http://cdna.eva.mpg.de/neandertal/Vindija/VCF/Denisova/1,2) We selected sites if the 3 ancient humans were similar to the hg19 references, the Denisovan genome was mutated and if at least 2 Neanderthals out of three were mutated for the base. From this criterion, we selected 171 sites.

**Position Weight Matrix search (PWM):**

From these sites, we extracted short sequences of 31bp (15bp before the mutated site, 15bp after) one for humans (hg19) and one for ancient hominins. Position Weight Matrix search scans was done with

MOODS-DNA ^53^ and the analysis of human matrixes with the JASPAR database ^54^. JASPAR [(http://jaspar.genereg.net)](http://jaspar.genereg.net/) is an open-access database of curated, non-redundant transcription factor (TF)-binding profiles stored as position frequency matrices (PFMs) for TFs across multiple species in six taxonomic groups to identify novel binding sites specific to humans and binding sites lost in extinct hominids ^54^. We used a threshold with pvalue <= 0.001. For each matrix and for each site, if we found a hit for human and not for ancient hominin we classified the hit as human novelty.

**Selection of brain-only transcription factors:**

From these scans, we extracted new sites of binding. We used the Human Protein Atlas in order to focus on transcription factors (202 genes) only expressed in the brain ^55^ [(https://www.proteinatlas.org/humanproteome/brain)](https://www.proteinatlas.org/humanproteome/brain).

**Analysis of single-cell genomics datasets:**

We took advantage of mouse, marmoset and human single-cell genomics datasets that are available to analyze AUTS2 gene expression in the adult cortex of these three species.

We took marmoset data from this URL: [https://cellxgene.cziscience.com/collections/0fd39ad7-5d2d41c2-bda0-c55bde614bdb](https://cellxgene.cziscience.com/collections/0fd39ad7-5d2d-41c2-bda0-c55bde614bdb) ^75^. We took the datasets related to cortex area (cortex.GLUT.all, cortex.GAD.all, cortex.OLIGO, cortex.ASTROCYTES).

We took mouse datasets from this URL: [https://allen-brain-cell-atlas.s3.us-west2.amazonaws.com/index.html#expression_matrices/WMB-10Xv3/20230630/](https://allen-brain-cell-atlas.s3.us-west-2.amazonaws.com/index.html#expression_matrices/WMB-10Xv3/20230630/2) ^76^. We took the dataset [WMB-10Xv3-CTXsp-raw](https://allen-brain-cell-atlas.s3-us-west-2.amazonaws.com/expression_matrices/WMB-10Xv3/20230630/WMB-10Xv3-CTXsp-raw.h5ad) and [WMB-10Xv3-Isocortex-1-raw.](https://allen-brain-cell-atlas.s3-us-west-2.amazonaws.com/expression_matrices/WMB-10Xv3/20230630/WMB-10Xv3-Isocortex-1-raw.h5ad) For the WMB-10xv3-Isocortex dataset we sub sampled 10,000 cells.

For all these datasets we applied the log-normalization (NormalizeData function) from the Seurat v4 R packages ^92^ and took the value of *AUTS2* gene (ENSMUSG00000029673 for mouse, ENSG00000158321 for the marmoset).

For the marmoset datasets we took the values of all cells. For mouse data we used the “class” annotation in order to get the AUTS2 expression value per cell type.

Single-cell genomics data for Human cortex from Velmeshev et al. ^71^ are available in UCSC genome browser webside (at [UCSC Genome Browser Home)](https://genome.ucsc.edu/).

We took Mouse E13.5 dataset from the Mouse Organogenesis Cell Atlas (MOCA) ^79^. We selected only cells at stage 13.5 and tagged as “neuronal” based on the main cell type metadata. We removed cells annotated as doublet. From that we selected 10000 cells randomly. We log-normalized and scaled the count data with the Seurat3 R package ^93^.

Human Data: We took the data from Velmeshev *et al* ^72^. We log-normalized and scaled the count data with the Seurat3 R package.

**Analysis of ENCODE enhancers:**

We took the ENCODE Candidate Cis-Regulatory Elements (cCREs) ^94^ from the UCSC genome browser website (at [UCSC Genome Browser Home)](https://genome.ucsc.edu/) and intersected with our “new in humans” transcription factors binding sites.

**Correlations and testing:**

For all datasets we ran Pearson’s correlation with R (cor function) for selected genes. Reported p-values are the p-values of the correlation test (cor.test function in R) for AUTS2 and the other genes. For multi testing issues we used Bonferroni correction and set the maximal significant p-value at 0.05/(number of tests).

## References

1. Sultana, R. *et al.* Identification of a novel gene on chromosome 7q11.2 interrupted by a translocation breakpoint in a pair of autistic twins. *Genomics* **80**, 129–134 (2002).

2. Gao, Z. *et al.* An AUTS2-Polycomb complex activates gene expression in the CNS. *Nature* **516**, 349–354 (2014).

3. Green, R. E. *et al.* A draft sequence of the Neandertal genome. *Science* **328**, 710–722 (2010).

4. Oksenberg, N., Stevison, L., Wall, J. D. & Ahituv, N. Function and regulation of AUTS2, a gene implicated in autism and human evolution. *PLoS Genet* **9**, e1003221 (2013).

5. Whalen, S. & Pollard, K. S. Enhancer Function and Evolutionary Roles of Human Accelerated Regions. *Annu Rev Genet* **56**, 423–439 (2022).

6. Beunders, G. *et al.* A detailed clinical analysis of 13 patients with AUTS2 syndrome further delineates the phenotypic spectrum and underscores the behavioural phenotype. *J Med Genet* **53**, 523–532 (2016).

7. Beunders, G. *et al.* Exonic deletions in AUTS2 cause a syndromic form of intellectual disability and suggest a critical role for the C terminus. *Am J Hum Genet* **92**, 210–220 (2013).

8. Elia, J. *et al.* Rare structural variants found in attention-deficit hyperactivity disorder are preferentially associated with neurodevelopmental genes. *Mol Psychiatry* **15**, 637–646 (2010).

9. Talkowski, M. E. *et al.* Sequencing chromosomal abnormalities reveals neurodevelopmental loci that confer risk across diagnostic boundaries. *Cell* **149**, 525–537 (2012).

10. Schumann, G. *et al.* Genome-wide association and genetic functional studies identify autism susceptibility candidate 2 gene (AUTS2) in the regulation of alcohol consumption. *Proc Natl Acad Sci U S A* **108**, 7119–7124 (2011).

11. Chen, Y.-H., Liao, D.-L., Lai, C.-H. & Chen, C.-H. Genetic analysis of AUTS2 as a susceptibility gene of heroin dependence. *Drug Alcohol Depend* **128**, 238–242 (2013).

12. Mefford, H. C. *et al.* Genome-wide copy number variation in epilepsy: novel susceptibility loci in idiopathic generalized and focal epilepsies. *PLoS Genet* **6**, e1000962 (2010).

13. Zhang, B. *et al.* Association study identifying a new susceptibility gene (AUTS2) for schizophrenia. *Int J Mol Sci* **15**, 19406–19416 (2014).

14. Ozsoy, F., Karakus, N. B., Yigit, S. & Kulu, M. Effect of AUTS2 gene rs6943555 variant in male patients with schizophrenia in a Turkish population. *Gene* **756**, 144913 (2020).

15. Girirajan, S. *et al.* Relative burden of large CNVs on a range of neurodevelopmental phenotypes. *PLoS Genet* **7**, e1002334 (2011).

16. Doust, C. *et al.* Discovery of 42 genome-wide significant loci associated with dyslexia. *Nat Genet* **54**, 1621–1629 (2022).

17. Biel, A. *et al.* AUTS2 Syndrome: Molecular Mechanisms and Model Systems. *Front Mol Neurosci* **15**, 858582 (2022).

18. Shimogori, T. *et al.* Digital gene atlas of neonate common marmoset brain. *Neurosci Res* **128**, 1–13 (2018).

19. Kita, Y. *et al.* Cellular-resolution gene expression profiling in the neonatal marmoset brain reveals dynamic species- and region-specific differences. *Proc Natl Acad Sci U S A* **118**, e2020125118 (2021).

20. Lepagnol-Bestel, A.-M. *et al.* SLC25A12 expression is associated with neurite outgrowth and is upregulated in the prefrontal cortex of autistic subjects. *Mol Psychiatry* **13**, 385–397 (2008).

21. Bedogni, F. *et al.* Autism susceptibility candidate 2 (Auts2) encodes a nuclear protein expressed in developing brain regions implicated in autism neuropathology. *Gene Expr Patterns* **10**, 9–15 (2010).

22. Yamashiro, K. *et al.* AUTS2 Governs Cerebellar Development, Purkinje Cell Maturation, Motor Function and Social Communication. *iScience* **23**, 101820 (2020).

23. Besnard, A. & Leroy, F. Top-down regulation of motivated behaviors via lateral septum sub-circuits. *Mol Psychiatry* **27**, 3119–3128 (2022).

24. Burguière, E., Monteiro, P., Mallet, L., Feng, G. & Graybiel, A. M. Striatal circuits, habits, and implications for obsessive-compulsive disorder. *Curr Opin Neurobiol* **30**, 59–65 (2015).

25. Josselyn, S. A. & Tonegawa, S. Memory engrams: Recalling the past and imagining the future. *Science* **367**, (2020).

26. Russo, S. J. & Nestler, E. J. The brain reward circuitry in mood disorders. *Nat Rev Neurosci* **14**, 609–625 (2013).

27. Dehaene, S., Cohen, L., Morais, J. & Kolinsky, R. Illiterate to literate: behavioural and cerebral changes induced by reading acquisition. *Nat Rev Neurosci* **16**, 234–244 (2015).

28. Raslau, F. D. *et al.* Memory part 2: the role of the medial temporal lobe. *AJNR Am J Neuroradiol* **36**, 846–849 (2015).

29. Hitti, F. L. & Siegelbaum, S. A. The hippocampal CA2 region is essential for social memory. *Nature* **508**, 88–92 (2014).

30. Middleton, S. J. & McHugh, T. J. CA2: A Highly Connected Intrahippocampal Relay. *Annu Rev Neurosci* **43**, 55–72 (2020).

31. Johansen, J. P., Cain, C. K., Ostroff, L. E. & LeDoux, J. E. Molecular mechanisms of fear learning and memory. *Cell* **147**, 509–524 (2011).

32. Smith, J. B., Lee, A. K. & Jackson, J. The claustrum. *Curr Biol* **30**, R1401–R1406 (2020).

33. Crick, F. C. & Koch, C. What is the function of the claustrum? *Philos Trans R Soc Lond B Biol Sci* **360**, 1271–1279 (2005).

34. Peng, H. *et al.* Morphological diversity of single neurons in molecularly defined cell types. *Nature* **598**, 174–181 (2021).

35. Livingstone, M. S., Rosen, G. D., Drislane, F. W. & Galaburda, A. M. Physiological and anatomical evidence for a magnocellular defect in developmental dyslexia. *Proc Natl Acad Sci U S A* **88**, 7943–7947 (1991).

36. Spiteri, S. & Crewther, D. Neural Mechanisms of Visual Motion Anomalies in Autism: A Two-Decade Update and Novel Aetiology. *Front Neurosci* **15**, 756841 (2021).

37. Charpak, G., Dominik, W. & Zaganidis, N. Optical imaging of the spatial distribution of beta-particles emerging from surfaces. *Proc Natl Acad Sci U S A* **86**, 1741–1745 (1989).

38. O’Rahilly, R. & Müller, F. Neurulation in the normal human embryo. *Ciba Found Symp* **181**, 70–82; discussion 82-89 (1994).

39. Silberberg, S. N. *et al.* Subpallial Enhancer Transgenic Lines: a Data and Tool Resource to Study Transcriptional Regulation of GABAergic Cell Fate. *Neuron* **92**, 59–74 (2016).

40. Nery, S., Fishell, G. & Corbin, J. G. The caudal ganglionic eminence is a source of distinct cortical and subcortical cell populations. *Nat Neurosci* **5**, 1279–1287 (2002).

41. Lim, L., Mi, D., Llorca, A. & Marín, O. Development and Functional Diversification of Cortical Interneurons. *Neuron* **100**, 294–313 (2018).

42. Hu, J. S. *et al.* Coup-TF1 and Coup-TF2 control subtype and laminar identity of MGE-derived neocortical interneurons. *Development* **144**, 2837–2851 (2017).

43. Bandler, R. C., Mayer, C. & Fishell, G. Cortical interneuron specification: the juncture of genes, time and geometry. *Curr Opin Neurobiol* **42**, 17–24 (2017).

44. Xu, Q., Tam, M. & Anderson, S. A. Fate mapping Nkx2.1-lineage cells in the mouse telencephalon. *J Comp Neurol* **506**, 16–29 (2008).

45. Lepagnol-Bestel, A. M. *et al.* AUTS2 gene dosage affects synaptic AMPA receptors via a local dendritic spine AUTS2-TTC3-AKT-mTORC1 signaling dysfunction. *bioRxiv* (2022).

46. Trujillo, C. A. *et al.* Reintroduction of the archaic variant of NOVA1 in cortical organoids alters neurodevelopment. *Science* **371**, eaax2537 (2021).

47. Pinson, A. *et al.* Human TKTL1 implies greater neurogenesis in frontal neocortex of modern humans than Neanderthals. *Science* **377**, eabl6422 (2022).

48. Meyer, M. *et al.* A high-coverage genome sequence from an archaic Denisovan individual. *Science* **338**, 222–226 (2012).

49. Prüfer, K. *et al.* The complete genome sequence of a Neanderthal from the Altai Mountains. *Nature* **505**, 43–49 (2014).

50. Prüfer, K. *et al.* A high-coverage Neandertal genome from Vindija Cave in Croatia. *Science* **358**, 655–658 (2017).

51. Lazaridis, I. *et al.* Ancient human genomes suggest three ancestral populations for present-day Europeans. *Nature* **513**, 409–413 (2014).

52. Fu, Q. *et al.* Genome sequence of a 45,000-year-old modern human from western Siberia. *Nature* **514**, 445–449 (2014).

53. Korhonen, J. H., Palin, K., Taipale, J. & Ukkonen, E. Fast motif matching revisited: high-order PWMs, SNPs and indels. *Bioinformatics* **33**, 514–521 (2017).

54. Castro-Mondragon, J. A. *et al.* JASPAR 2022: the 9th release of the open-access database of transcription factor binding profiles. *Nucleic Acids Res* **50**, D165–D173 (2022).

55. Sjöstedt, E. *et al.* An atlas of the protein-coding genes in the human, pig, and mouse brain. *Science* **367**, eaay5947 (2020).

56. Bulfone, A. *et al.* T-brain-1: a homolog of Brachyury whose expression defines molecularly distinct domains within the cerebral cortex. *Neuron* **15**, 63–78 (1995).

57. Bedogni, F. *et al.* Tbr1 regulates regional and laminar identity of postmitotic neurons in developing neocortex. *Proc Natl Acad Sci U S A* **107**, 13129–13134 (2010).

58. Fazel Darbandi, S. *et al.* Neonatal Tbr1 Dosage Controls Cortical Layer 6 Connectivity. *Neuron* **100**, 831-845.e7 (2018).

59. Joyner, A. L., Herrup, K., Auerbach, B. A., Davis, C. A. & Rossant, J. Subtle cerebellar phenotype in mice homozygous for a targeted deletion of the En-2 homeobox. *Science* **251**, 1239–1243 (1991).

60. Genestine, M. *et al.* Engrailed-2 (En2) deletion produces multiple neurodevelopmental defects in monoamine systems, forebrain structures and neurogenesis and behavior. *Hum Mol Genet* **24**, 5805–5827 (2015).

61. Saunders, A. *et al.* Molecular Diversity and Specializations among the Cells of the Adult Mouse Brain. *Cell* **174**, 1015-1030.e16 (2018).

62. Ooi, L. & Wood, I. C. Chromatin crosstalk in development and disease: lessons from REST. *Nat Rev Genet* **8**, 544–554 (2007).

63. Loe-Mie, Y. *et al.* SMARCA2 and other genome-wide supported schizophrenia-associated genes: regulation by REST/NRSF, network organization and primate-specific evolution. *Hum. Mol. Genet.* **19**, 2841–2857 (2010).

64. Zou, Z., Ohta, T., Miura, F. & Oki, S. ChIP-Atlas 2021 update: a data-mining suite for exploring epigenomic landscapes by fully integrating ChIP-seq, ATAC-seq and Bisulfite-seq data. *Nucleic Acids Res* **50**, W175–W182 (2022).

65. Buenrostro, J. D., Giresi, P. G., Zaba, L. C., Chang, H. Y. & Greenleaf, W. J. Transposition of native chromatin for multimodal regulatory analysis and personal epigenomics. *Nat Methods* **10**, 1213–1218 (2013).

66. Pollard, K. S. *et al.* Forces Shaping the Fastest Evolving Regions in the Human Genome. *PLoS Genet* **2**, e168 (2006).

67. Prabhakar, S., Noonan, J. P., Pääbo, S. & Rubin, E. M. Accelerated Evolution of Conserved Noncoding Sequences in Humans. *Science* (2006) doi:10.1126/science.1130738.

68. Pinto, D. *et al.* Functional Impact of Global Rare Copy Number Variation in Autism Spectrum Disorder. *Nature* **466**, 368–372 (2010).

69. Capra, J. A., Erwin, G. D., McKinsey, G., Rubenstein, J. L. R. & Pollard, K. S. Many human accelerated regions are developmental enhancers. *Philos Trans R Soc Lond B Biol Sci* **368**, 20130025 (2013).

70. Burbano, H. A. *et al.* Analysis of Human Accelerated DNA Regions Using Archaic Hominin Genomes. *PLoS One* **7**, e32877 (2012).

71. Velmeshev, D. *et al.* Single-cell genomics identifies cell type–specific molecular changes in autism. *Science* **364**, 685–689 (2019).

72. Velmeshev, D. *et al.* Single-cell analysis of prenatal and postnatal human cortical development. *Science* **382**, eadf0834 (2023).

73. Arenas, E., Denham, M. & Villaescusa, J. C. How to make a midbrain dopaminergic neuron. *Development* **142**, 1918–1936 (2015).

74. Peterson, D. J. *et al.* The Basic Helix-Loop-Helix Gene Nato3 Drives Expression of Dopaminergic Neuron Transcription Factors in Neural Progenitors. *Neuroscience* **421**, 176–191 (2019).

75. Krienen, F. M. *et al.* A marmoset brain cell census reveals regional specialization of cellular identities. *Sci Adv* **9**, eadk3986.

76. Zhang, M. *et al.* Molecularly defined and spatially resolved cell atlas of the whole mouse brain. *Nature* **624**, 343–354 (2023).

77. Zullo, J. M. *et al.* Regulation of Lifespan by Neural Excitation and REST. *Nature* **574**, 359–364 (2019).

78. Prestigio, C. *et al.* REST/NRSF drives homeostatic plasticity of inhibitory synapses in a target-dependent fashion. *eLife* **10**, e69058.

79. Cao, J. *et al.* The single cell transcriptional landscape of mammalian organogenesis. *Nature* **566**, 496–502 (2019).

80. Dölen, G. *et al.* Pathophysiological Toolkit: Genes to Circuits. in *Translational Neuroscience: Toward New Therapies* (eds. Nikolich, K. & Hyman, S. E.) (MIT Press, Cambridge (MA), 2015).

81. Hori, K. *et al.* Heterozygous Disruption of Autism susceptibility candidate 2 Causes Impaired Emotional Control and Cognitive Memory. *PLoS One* **10**, e0145979 (2015).

82. Hori, K. *et al.* AUTS2 Regulation of Synapses for Proper Synaptic Inputs and Social Communication. *iScience* **23**, 101183 (2020).

83. Li, J. *et al.* Auts2 deletion involves in DG hypoplasia and social recognition deficit: The developmental and neural circuit mechanisms. *Sci Adv* **8**, eabk1238 (2022).

84. Engmann, O. *et al.* Cocaine-Induced Chromatin Modifications Associate With Increased Expression and Three-Dimensional Looping of Auts2. *Biol Psychiatry* **82**, 794–805 (2017).

85. Misra, V. The social brain network and autism. *Ann Neurosci* **21**, 69–73 (2014).

86. Corbin, J. G., Nery, S. & Fishell, G. Telencephalic cells take a tangent: non-radial migration in the mammalian forebrain. *Nat Neurosci* **4 Suppl**, 1177–1182 (2001).

87. Stickels, R. R. *et al.* Highly sensitive spatial transcriptomics at near-cellular resolution with Slide-seqV2. *Nat Biotechnol* **39**, 313–319 (2021).

88. Chen, K. H., Boettiger, A. N., Moffitt, J. R., Wang, S. & Zhuang, X. Spatially resolved, highly multiplexed RNA profiling in single cells. *Science* **348**, aaa6090 (2015).

89. Janesick, A. *et al.* High resolution mapping of the tumor microenvironment using integrated single-cell, spatial and in situ analysis. *Nat Commun* **14**, 8353 (2023).

90. Yao, Z. *et al.* A high-resolution transcriptomic and spatial atlas of cell types in the whole mouse brain. *Nature* **624**, 317–332 (2023).

91. Okhovat, M. *et al.* TAD evolutionary and functional characterization reveals diversity in mammalian TAD boundary properties and function. *Nat Commun* **14**, 8111 (2023).

92. Hao, Y. *et al.* Integrated analysis of multimodal single-cell data. *Cell* **184**, 3573-3587.e29 (2021).

93. Hao, Y. *et al.* Dictionary learning for integrative, multimodal, and massively scalable single-cell analysis. *Nat Biotechnol* **42**, 293–304 (2024).

94. Moore, J. E. *et al.* Expanded encyclopaedias of DNA elements in the human and mouse genomes. *Nature* **583**, 699–710 (2020).
